# Unveiling mycoviral diversity in *Ophiocordyceps sinensis* through transcriptome analyses

**DOI:** 10.3389/fmicb.2024.1493365

**Published:** 2024-11-25

**Authors:** Qin Kang, Jihong Zhang, Fangzhou Chen, Caihong Dong, Qilian Qin, Xuan Li, Hongtuo Wang, Huan Zhang, Qian Meng

**Affiliations:** ^1^State Key Laboratory of Integrated Management of Pest Insects and Rodents, Institute of Zoology, Chinese Academy of Sciences, Beijing, China; ^2^University of Chinese Academy of Sciences, Beijing, China; ^3^Beijing Yun’an Bio-tech Co. Ltd., Beijing, China; ^4^China Pharmaceutical University, School of Pharmacy, Nanjing, China; ^5^State Key Laboratory of Mycology, Institute of Microbiology, Chinese Academy of Sciences, Beijing, China

**Keywords:** *Ophiocordyceps sinensis*, Mycovirus, transcriptome, public transcriptome mining, viral taxonomy

## Abstract

*Ophiocordyceps sinensis*, an entomopathogenic fungus, infects larvae from the Lepidoptera: Hepialidae family, forming the valuable Chinese cordyceps. Mycoviruses are widespread across major lineages of filamentous fungi, oomycetes, and yeasts and have the potential to influence fungal biology and ecology. This study aimed to detect mycovirus within *O. sinensis* by isolating double-stranded RNA from six stains for transcriptomic sequencing and analyzing publicly available transcriptome data from 13 *O. sinensis* representative samples. Our analysis revealed 13 mycoviruses, with nine reported for the first time in *O. sinensis*. These mycoviruses are distributed across five families—*Partitiviridae*, *Mitoviridae*, *Narnaviridae*, *Botourmiaviridae*, *Deltaflexiviridae*—and two unclassified lineages, Ormycovirus and Vivivirus. This study also revealed frequent coinfections within individual *O. sinensis* strains and dynamic shifts in viral composition during fungal development. These findings enhance our knowledge of mycovirus diversity within *O. sinensis* and provide new insights into their taxonomy.

## 1 Introduction

Chinese cordyceps is an insect-fungus complex generated by *Ophiocordyceps sinensis* (*Ophiocordyceps*, Ophiocordycipitaceae, Hypocreales, Sordariomycetes, Ascomycota) infecting insect larvae (Lepidoptera, Hepialidae). Beyond its natural soil habitat, *O. sinensis* can endogenously colonize plants (Baral, 2017). Chinese cordyceps is widely distributed across the alpine grasslands of the Himalayas and the mountainous regions of the Tibetan Plateau (Zhang et al., 2012). As a traditional Chinese medicine for centuries, Chinese cordyceps has become one of the most expensive natural products, sometimes surpassing gold in price, and is key to the local economy (Fan and He, 2024). Artificial cultivation of Chinese cordyceps is an effective way to alleviate supply and demand pressures and ecological impacts. However, technological bottlenecks still exist, such as low natural infection rates, long production cycles, and disease susceptibility caused by other pathogens (Qin et al., 2018).

Mycoviruses infect and replicate within fungi. They have been increasingly identified since the first discovery in *Agaricus bisporus* in 1962 (Hollings, 1962). Advances in high throughput sequencing (HTS) have facilitated the identification of mycoviruses in various fungal species, leading to a substantial increase in the number of described viruses infecting fungi in recent years (Villan Larios et al., 2023). Most mycoviruses have genomes consisting of positive-sense (+) or negative-sense (−) single-stranded RNA (ssRNA), double-stranded RNA (dsRNA) or even DNA (Sato and Suzuki, 2023). Currently, mycoviruses can be identified via dsRNA analysis and HTS. With the accessibility of fungal transcriptome data on platforms like NCBI Sequence Read Archive (SRA), the presence of mycoviruses in less-studied fungal populations can be explored (Hough et al., 2023). Although most mycovirus infections are asymptomatic, certain mycoviruses can influence various aspects of fungal physiology, including growth rate, reproduction modes, adaptability, and pathogenicity (Kotta-Loizou, 2021; Sato and Suzuki, 2023). In entomopathogenic fungi, particularly in the *Metarhizium* and *Beauveria bassiana*, mycoviruses have been relatively well-documented (Filippou et al., 2018; Hwang et al., 2023; Guo et al., 2024; Shi et al., 2024). In recent years, three different viruses—Cordyceps chanhua alternavirus 1 (CcAV1), Cordyceps chanhua victorivirus 1 (CcV1), and Cordyceps chanhua partitivirus 1 (CchPV1)—have been identified within *Cordyceps chanhua*. CchPV1 has been shown to affect development and multi-stress tolerance in the host fungus (Zhang et al., 2022; Zhu et al., 2022a; Zhu et al., 2022b). However, research on mycoviruses in *O. sinensis* remains limited.

While residing in extreme ecosystems, *O. sinensis* interacts with insects, plants, and other microorganisms, where mycoviruses likely influence its development and prevalence. Early studies mining public databases suggest the existence of mycoviruses in *O. sinensis*, but systematic investigation is lacking (Forgia et al., 2022; Gilbert et al., 2019). To address this gap, we selected six rapidly growing *O. sinensis* strains from our laboratory for dsRNA screening and transcriptomic sequencing. Additionally, we analyzed publicly available *O. sinensis* transcriptome data in NCBI SRA to assess the diversity of mycoviruses. Our findings provide valuable insights into the diversity, origins and evolutionary relationships of mycoviruses in *O. sinensis*, as well as their genomic structure and potential functions.

## 2 Materials and methods

### 2.1 Isolates and growth conditions

Strains IOZ14 and IOZ23 were isolated from sclerotium samples of *O. sinensis*, collected in Guoluo Tibetan Autonomous Prefecture, Qinghai Province, China. Strains IOZ25, IOZ26, and IOZ31 were derived from fresh Chinese cordyceps harvested in Xiaojin County, Sichuan Province, China, while strain IOZ29 was derived from fresh Chinese cordyceps collected in Yushu Tibetan Autonomous Prefecture, Qinghai Province, China. *O. sinensis* was cultured in peptone potato dextrose agar (PPDA) medium at 18°C to harvest conidia and mycelium, following previously described methods (Wu et al., 2022a).

### 2.2 Extraction and screening of dsRNA

After 1 month of cultivation, fresh mycelium samples were collected for nucleic acid extraction. The dsRNA was extracted using the CF11 cellulose powder adsorption method (Morris and Dodds, 1979), and then digested by S1 nuclease and DNase I (TaKaRa, Dalian, China) to remove DNA and ssRNA. The purified dsRNA was analyzed on a 1% agarose gel to detect mycovirus presence.

### 2.3 Extraction of total RNA and sequencing

Total RNA was extracted using TRIzol reagent (Thermo Fisher Scientific, Waltham, USA) following the manufacturer’s instructions, and then treated with DNase I (TaKaRa, Dalian, China) to eliminate genomic DNA. Two μg of RNA from each IOZ strain was pooled and sent to Novogene Company (Beijing, China) for library construction (using ribosomal RNA deletion method) and sequencing on the Illumina MiSeq 2000/2500.

### 2.4 Assembly and annotation of mycovirus sequences

The raw sequencing data were processed using fastp (version 0.23.2) with default parameters to filter out low-quality reads and adapter sequences (Chen et al., 2018). The resulting clean data were aligned to the *O. sinensis* genome (GCA_012934285.1) using HISAT2 (Kim et al., 2019). Subsequently, the unmapped reads were extracted and assembled *de novo* using Megahit (v1.2.9) with default parameters (Li D. et al., 2016). After removing redundancy, the assembled sequences were annotated by the Non-Redundant Protein Sequence Database (nr) database of NCBI to identify partial mycovirus sequences. To calculate the number of reads for the individually identified viruses in each sample, the clean reads were mapped to the final fragments of each virus using samtools (v1.20) (Danecek et al., 2021). The coverage of each mycovirus identified in the public transcriptome was calculated based on the length of the longest contig assembled for each mycovirus in the sample as a percentage of the length of the final viral genome length.

### 2.5 Reverse-transcription PCR (RT-PCR) and mycovirus full-length determination

To further confirm the presence of mycoviruses in the IOZ strains, reverse transcriptase M-MLV (TaKaRa, Dalian, China) and random primers were used to synthesize first-strand cDNA from 500 ng of RNA. Virus detection was carried out through PCR amplification using TransStart FastPfu DNA Polymerase (TransGen, Beijing, China). Primers specific for individual mycoviruses were designed based on the assembled sequences (Supplementary Table S1). The rapid amplification of cDNA ends (RACE) technique was employed to determine the complete sequences of all mycoviruses infecting the IOZ strain (Lambden et al., 1992). The primers used in the RACE analysis are listed in Supplementary Table S2.

### 2.6 Mining publicly available *Ophiocordyceps sinensis* transcriptomic datasets

The search term “(*Ophiocordyceps sinensis*) AND bioproject_sra[filter] NOT bioproject_gap[filter]” and “(Chinese [All Fields] AND (“Cordyceps”[Organism] OR cordyceps [All Fields])) AND “bioproject sra”[Filter]” were used to retrieve all available *O. sinensis* raw read files from the NCBI SRA database accessed on March 2, 2024^1^. Initially, a total of 64 bioprojects were returned. Through manual curation, samples were refined based on specific criteria, including the combination of samples from the same strain, exclusion of mixed RNA samples, and removal of samples with short or unavailable sequences. Data for further analysis were randomly drawn from one biological replicate of each control sample or wild-type strain. Additionally, different developmental stages of the same strain of Chinese cordyceps were also analyzed for virus screening.

### 2.7 Mycoviral genome and phylogenetic analysis

Open reading frames (ORFs) within the viral sequences were determined using NCBI ORF Finder program^2^. Conserved structural domains were predicted using NCBI Conserved Domain Search (CD-Search) tool^3^. RNA dependent RNA polymerase (RdRp) protein sequences were retrieved from NCBI database, followed by multiple sequence comparisons using MUSCLE (Edgar, 2022). Subsequently, the sequences were trimmed by trimAl (v1.4.rev15) with the parameters of gt 0.6 and cons 60 (Capella-Gutiérrez et al., 2009). Maximum likelihood phylogenetic analysis was performed using IQ-TREE 2.1.2, with 1,000 bootstrap replicates (Minh et al., 2020). The phylogenetic tree was visualized using MEGA 11 (Tamura et al., 2021).

## 3 Results

### 3.1 DsRNA screening

**Figure 1:**
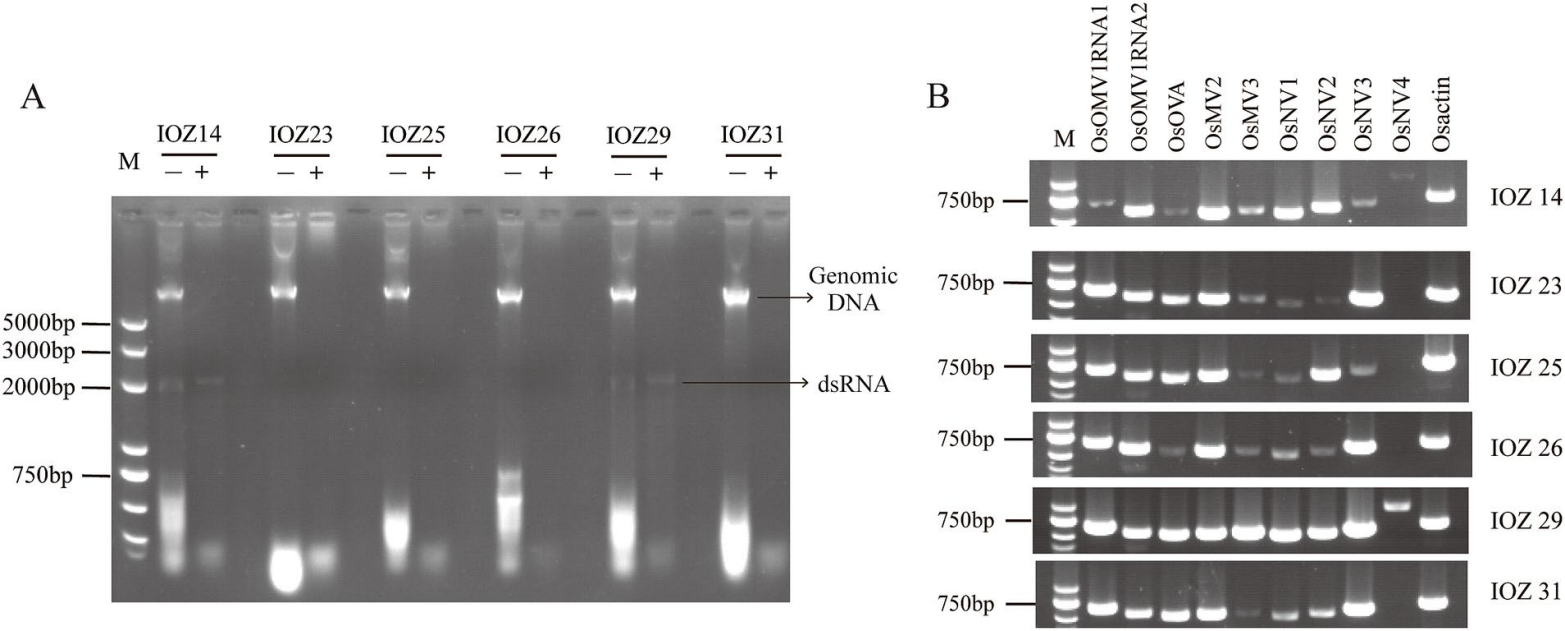
Detection of viruses in IOZ strains. (A) Detection of total viral dsRNAs in the IOZ strains. The dsRNA samples with (+) or without (−) DNase I and S1 nuclease treatment were analyzed on a 1% agarose gel. (B) RT-PCR amplification detection of viruses in IOZ strains. *O. sinensis actin* (Osactin) was used as the reference gene. OsOMV1RNA, RNA of Ophiocordyceps sinensis ormycovirus 1; OsOVA, Ophiocordyceps ourmiavirus A; OsMV, Ophiocordyceps sinensis mitovirus; OsNV, Ophiocordyceps sinensis narnavirus.

Six IOZ strains were screened for the presence of dsRNA molecules. At least one dsRNA fragment with a size between 2.0–3.0 kilobase (kb) was detected in IOZ14 and IOZ29 treated with DNase I and S1 nuclease (Figure 1A), indicating the existence of mycoviruses in these two strains. The fragment may be the genome of dsRNA viruses or the replicative form of single-stranded (ss) RNA viruses. Considering that the titers of certain RNA viruses are too low to detect (Kondo et al., 2022), a pooled sample of the six strains was subjected to RNA sequencing.

### 3.2 Viral sequences identified in the transcriptome of *Ophiocordyceps sinensis*

Transcriptomic sequencing of the IOZ stains yielded 36,302,134 raw reads. After quality filtering, a total of 32,038,562 clean reads remained, which were assembled into 70 contigs. The contigs that showed greater than 90% similarity with the reference virus at the amino acid level were considered to be the homology. The RT-PCR analyses showed that strains IOZ14 and IOZ29 contained eight viruses, while the other four strains each had seven viruses (Figure 1B). All of the detected viruses were (+) ssRNA viruses. Three of them were reported before in *O. sinensis* and the others were newly identified. They belong to four distinct taxa: *Botourmiaviridae*, *Mitoviridae*, *Narnaviridae* and Ormycovirus.

### 3.3 Viral sequences identified from publicly available *Ophiocordyceps sinensis* transcriptomes

Querying the NCBI SRA database for *O. sinensis* transcriptomic datasets initially returned 64 BioProjects. After manual screening, there are 13 SRA samples left for further analysis (Table 1). A total of 101 contigs were assembled from the 13 SRA samples and 13 viruses were identified (Supplementary Table S3). The number of reads and coverage validated the presence of each identified virus (Supplementary Table S4). These viruses included the eight identified in our transcriptome analysis, plus five others: Ophiocordyceps sinensis mitovirus 1 (OsMV1) and four novel viruses. Ophiocordyceps sinensis ormycovirus 1 (OsOMV1) was relatively the most prevalent, found in 8 SRA samples. Following closely were Ophiocordyceps ourmiavirus A (OsOVA), Ophiocordyceps sinensis mitovirus 3 (OsMV3) and Ophiocordyceps sinensis narnavirus 2 (OsNV2), each of which appeared in 5 samples. Ophiocordyceps sinensis narnavirus 3 (OsNV3), Ophiocordyceps sinensis partitivirus 1 (OsPV1) and Ophiocordyceps sinensis vivivirus 1 (OsVV1) were each identified in only a single, distinct sample (Table 2).

**Table 1:** Summary of *O. sinensis* datasets retrieved from the NCBI SRA database.

SRA ID	Bioproject	Strain	Collecting place	Tissue	Citation for data/Institute submitting an unpublished dataset	Number of mycovirus contigs predicted
SRR11548640	PRJNA625214	IOZ07	Xiaojin county, Sichuan Province, China	Cultured mycelia	Li et al. (2020)	2
SRR12952889	PRJNA673413	SICC 5.02	Chengdu Eastern Sunshine Co. Ltd	Mycoparasite complex	Zhang et al. (2021)	15
SRR13286702	PRJNA687052	GZC	NA	NA	Lanzhou Jiaotong University	3
SRR21290686	PRJNA874172	TOS-1	Zaduo County, Qinghai Province, China	Cultured mycelia	Tang et al. (2022)	8
SRR2533613	PRJNA292632	Strain 1,229	Guoluo, Qinghai, China	Cultured mycelia	Li Y. et al. (2016)	3
SRR3658815	PRJNA325365	NA	Qumalai county, Qinghai Province, China	NA	Shanghai Institute of Biological Sciences	2
SRR3658816	PRJNA325365	NA	Gonghe county, Qinghai Province, China	NA	Shanghai Institute of Biological Sciences	9
SRR3658817	PRJNA325365	NA	Zaduo county, Qinghai Province, China	NA	Shanghai Institute of Biological Sciences	7
SRR5282569	PRJNA376530	CGMCC 3.14243	NA	Asexual mycelium	Tong et al. (2020)	4
SRR5428527	PRJNA382001	NA	Nyingchi District of Tibet, China	NA	Xia et al. (2017)	15
SRR5446809	PRJNA382742	QHZD	NA	Cultured mycelia	Sun Yat-sen university	7
SRR8258357	PRJNA507459	NA	Sunshine Lake Pharma Co. Ltd.	Fruit body	Li et al. (2019)	19
SRR9290661	PRJNA548647	TZTR02	NA	Ascospores	Chongqing Academy of Chinese Material Medica	7

NA indicates that the data is not available or does not exist.

**Table 2:** Summary of mycoviral genomes identified from *O. sinensis* transcriptomic datasets.

	OsOMV1	OsOVA	OsMV1	OsMV2	OsMV3	OsMV4	OsNV1	OsNV2	OsNV3	OsNV4	OsPV1	OsVV1	OsDFV1
SRR11548640				+ (934)	+ (2113)								
SRR12952889	+ (14)	+ (69)			+ (134)		+ (49)						+ (16376)
SRR13286702	+ (28)												
SRR21290686	+ (900)			+ (4)	+ (221)			+ (85)					
SRR2533613			+ (1190)	+ (3104)									
SRR3658815	+ (14)							+ (173)					
SRR3658816		+ (25)	+ (188)	+ (4582)	+ (1038)		+ (3784)	+ (141)					
SRR3658817	+ (38)							+ (97)					
SRR5282569		+ (1630)				+ (37)							
SRR5428527	+ (552)					+ (797)		+ (472)			+ (178)		
SRR5446809	+ (19)												
SRR8258357	+ (27)	+ (110)				+ (13)	+ (21)			+ (22)		+ (29)	+ (1725)
SRR9290661		+ (12)			+ (12)	+ (22)			+ (28)	+ (362)			
Total number	8	5	2	4	5	4	3	5	1	2	1	1	2

OsOMV1, Ophiocordyceps sinensis ormycovirus 1; OsOVA, Ophiocordyceps ourmiavirus A; OsMV, Ophiocordyceps sinensis mitovirus; OsNV, Ophiocordyceps sinensis narnavirus; OsPV1, Ophiocordyceps sinensis partitivirus 1; OsVV1, Ophiocordyceps sinensis vivivirus 1; OsDFV1, Ophiocordyceps sinensis deltaflexivirus 1. The ‘+’ indicates the presence of mycovirus in the sample and the number in parentheses represents the number of reads for each mycovirus in the sample.

### 3.4 One virus characterized in the Ormycovirus

**Figure 2:**
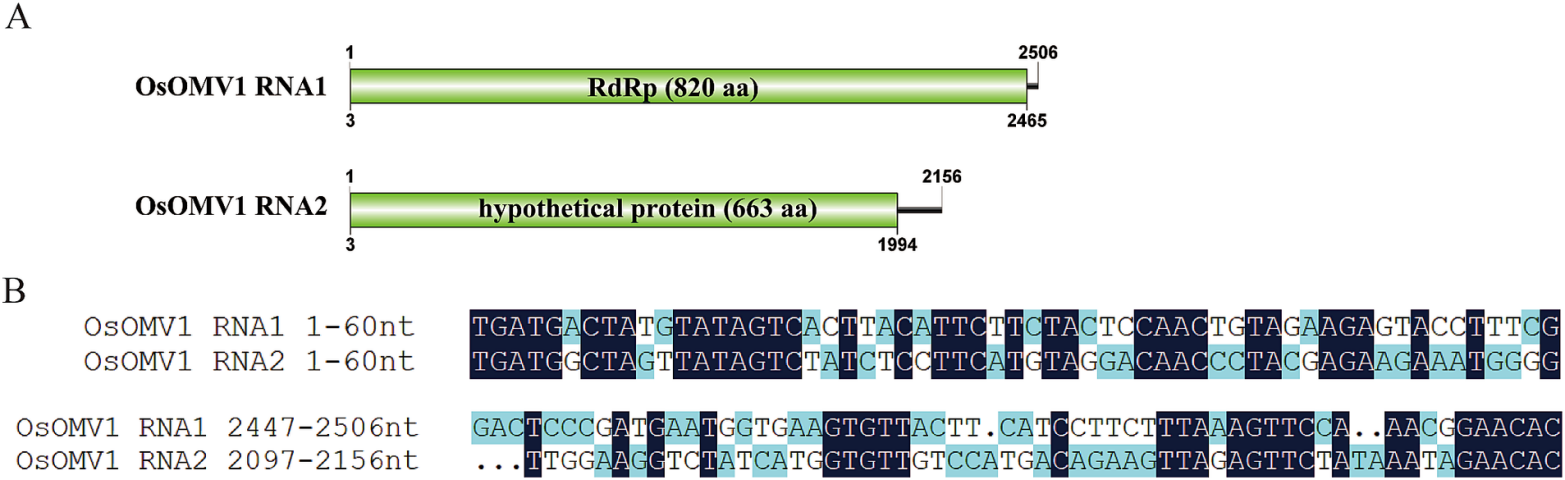
Genome organization and terminal conservation of OsOMV1. (A) Genomic structure of OsOMV1. ORFs are shown as boxes. (B) Terminal conservation between OsOMV1 RNA1 and RNA2 is shown by sequence alignment.

Ormycovirus is a newly defined group of viruses that contain two RNA fragments. The RNA1 encodes an RdRP, while the RNA2 encodes a putative protein (Pagnoni et al., 2023). By utilizing tBLASTn analysis against the NCBI available fungal Transcriptome Shotgun Assembly (TSA) databases, ormycovirus was identified in *O. sinensis* and classified within the group betormycovirus, named Ophiocordyceps sinensis ormycovirus 1 (OsOMV1) (Forgia et al., 2022). The OsOMV1 sequences, previously incomplete in the public databases, were fully characterized in this study using RACE. RNA1 and RNA2 of OsOMV1 were determined to be 2,506nt and 2,156nt long, respectively (Figure 2A). The RNA1 was predicted to encode an RdRp of 820 aa, sharing 43.7% sequence identity with the RdRp encoded by Erysiphe lesion-associated ormycovirus 2 (query coverage: 88%; *E*-value: 0; and accession: USW07207.1). The RNA2 appeared to encode a 663-aa protein, displaying 36.0% identity with that of Verticillium dahliae ormycovirus (query coverage: 71%; *E*-value: 3e^−93^; and accession: WPV08071.1). Both of the 5′ and 3′ termini in the two RNA fragments are highly conserved (Figure 2B). In addition, the terminal regions exhibited a high similarity to those of Starmerella bacillaris ormycovirus 1 (SbOMV1) (Forgia et al., 2022).

### 3.5 One virus characterized in the family *Botourmiaviridae*

**Figure 3:**
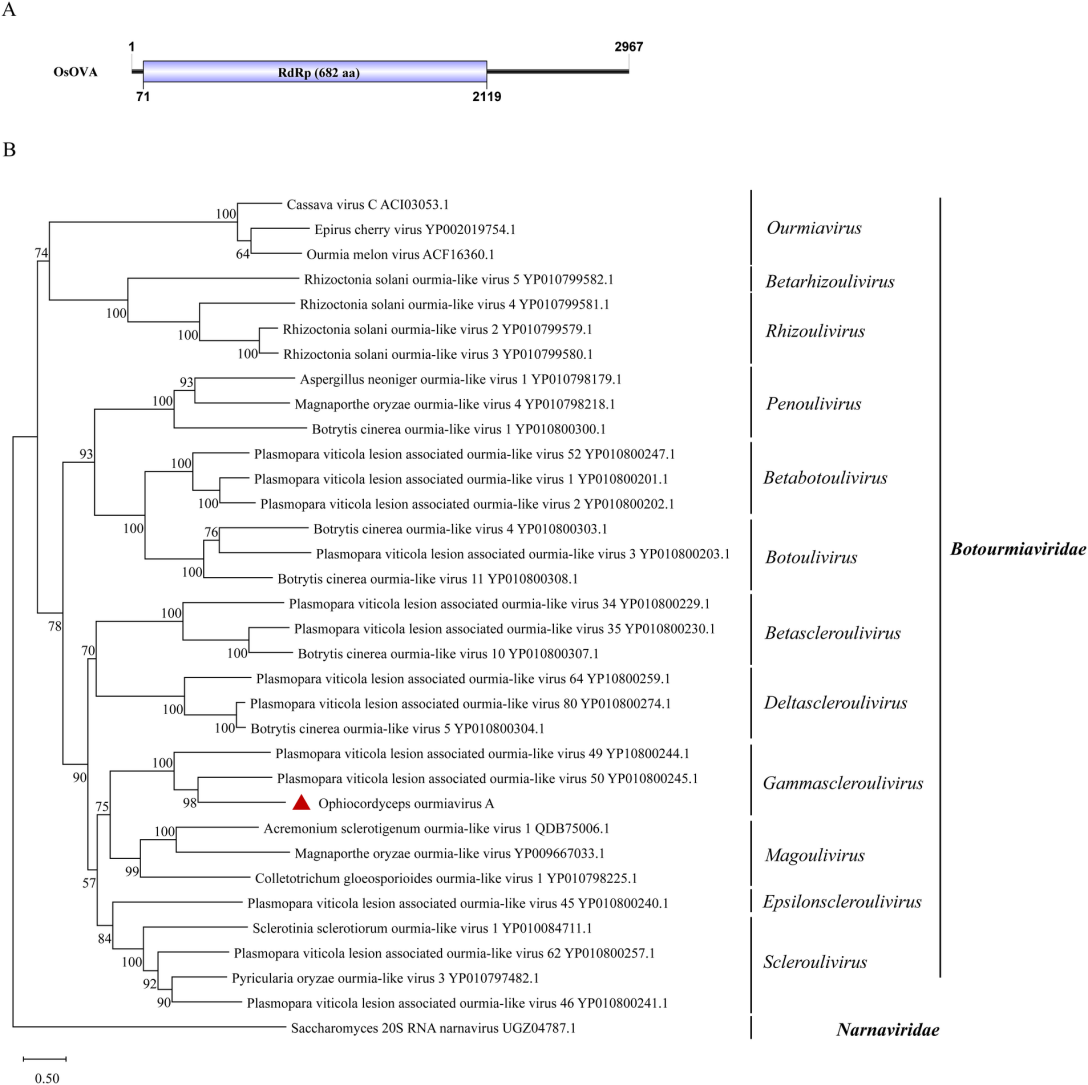
Genome organization and phylogenetic analysis of OsOVA. (A) Genomic structure of OsOVA. ORF is shown as a box. (B) Evolutionary relationships of the viruses in the *Botourmiaviridae* family. RdRp amino acid sequences from different genera of *Botourmiaviridae* were aligned to derive a phylogenetic tree using the maximum likelihood method with the LG + I + G substitution model. *Narnaviridae* is set as the outgroup. Bootstrap values (%) of 1,000 replicates that are greater than 50% are labeled on the branches. The virus OsOVA is marked with a red triangle.

Viruses of the family *Botourmiaviridae* are capable of infecting plants and filamentous fungi. *Botourmiaviridae* encompass 12 genera with (+) ssRNA genomes that can be either single or multiple segments (Ayllón et al., 2020). A virus from this family has been identified in *O. sinensis*. The viral partial sequence has been deposited in the NCBI database and designated as Ophiocordyceps ourmiavirus A (OsOVA). Its complete genome sequence was obtained through RACE. Notably, the OsOVA possesses a single segment with a full length of 2,967 nt, encoding a 682-aa RdRp (Figure 3A). The nucleotide sequence showed 51.0% identity with the RdRp sequence from Hulunbuir botou tick virus 5 (query coverage: 67%; *E*-value: 0; and accession: UYL95443.1). Phylogenetic analysis indicated that OsOVA exhibits a close relationship with Plasmopara viticola lesion associated ourmia-like virus 49, and 50, both belonging to the genus *Gammascleroulivirus* (Figure 3B).

### 3.6 Three viruses characterized in the family *Mitoviridae*

**Figure 4:**
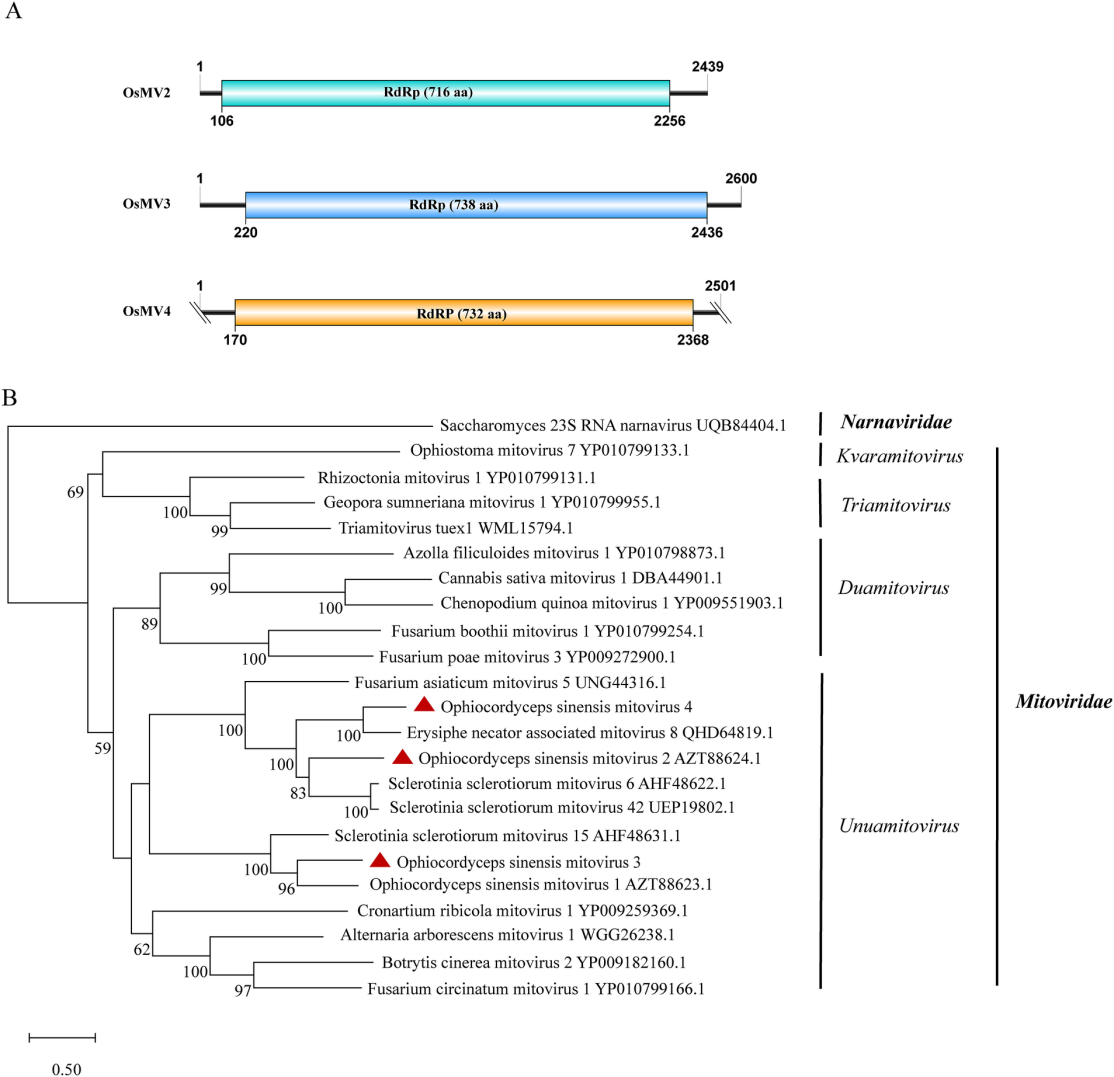
Genome organization and phylogenetic analysis of OsMV2–4. (A) Genomic structures of OsMV2–4. ORFs are shown as boxes. (B) Evolutionary relationships of the viruses in the *Mitoviridae* family. RdRp amino acid sequences from different genera of *Mitoviridae* were aligned to derive a phylogenetic tree using the maximum likelihood method with the LG + I + G substitution model. *Narnaviridae* is set as the outgroup. Bootstrap values (%) of 1,000 replicates that are greater than 50% are labeled on the branches. The viruses OsMV2–4 detected in this study are marked with a red triangle.

*Mitoviridae*, a family of capsid-less (+) ssRNA viruses, associates with the mitochondria of fungi, plants or invertebrates. Viruses in the family are predicted to harbor a single continuous ORF adhering to the mitochondrial translation system, where UGA encodes tryptophan (Begeman et al., 2023). Gilbert et al. previously identified Ophiocordyceps sinensis mitovirus 1 and 2 (OsMV1, OsMV2) through analysis of data available in NCBI SRA database (Gilbert et al., 2019). We detected two mitoviral genomes in the IOZ strains. One corresponded to OsMV2, while the other represented a novel mitovirus, designated as Ophiocordyceps sinensis mitovirus 3 (OsMV3). The complete genome of OsMV3 spans 2,600 nt and encodes a 738-aa RdRp protein (Figure 4A). The BLASTx analysis revealed 50.5% identity between the RdRp of OsMV3 and that of OsMV1 (query coverage: 83%; E-value: 0; and accession: AZT88623.1).

Additionally, we identified another novel mitovirus in samples SRR5282569, SRR5428527, SRR8258357 and SRR9290661 and named it Ophiocordyceps sinensis mitovirus 4 (OsMV4). The viral length is 2,501 nt which encodes a 732-aa RdRp protein. The completeness of the terminal sequence of OsMV4 is currently uncertain. BLASTx analysis showed an identity of 60.9% between the RdRp of OsMV4 and that of Erysiphe necator-associated mitovirus 8 (query coverage: 85%; E-value: 0; and accession: QHD64819.1). According to the International Committee on Taxonomy of Viruses (ICTV) classification, there are four genera in the family *Mitoviridae*. Phylogenetic analysis suggested that OsMV1-4 belong to the genus *Unuamitovirus* (Figure 4B).

### 3.7 Four novel viruses in the family *Narnaviridae*

**Figure 5:**
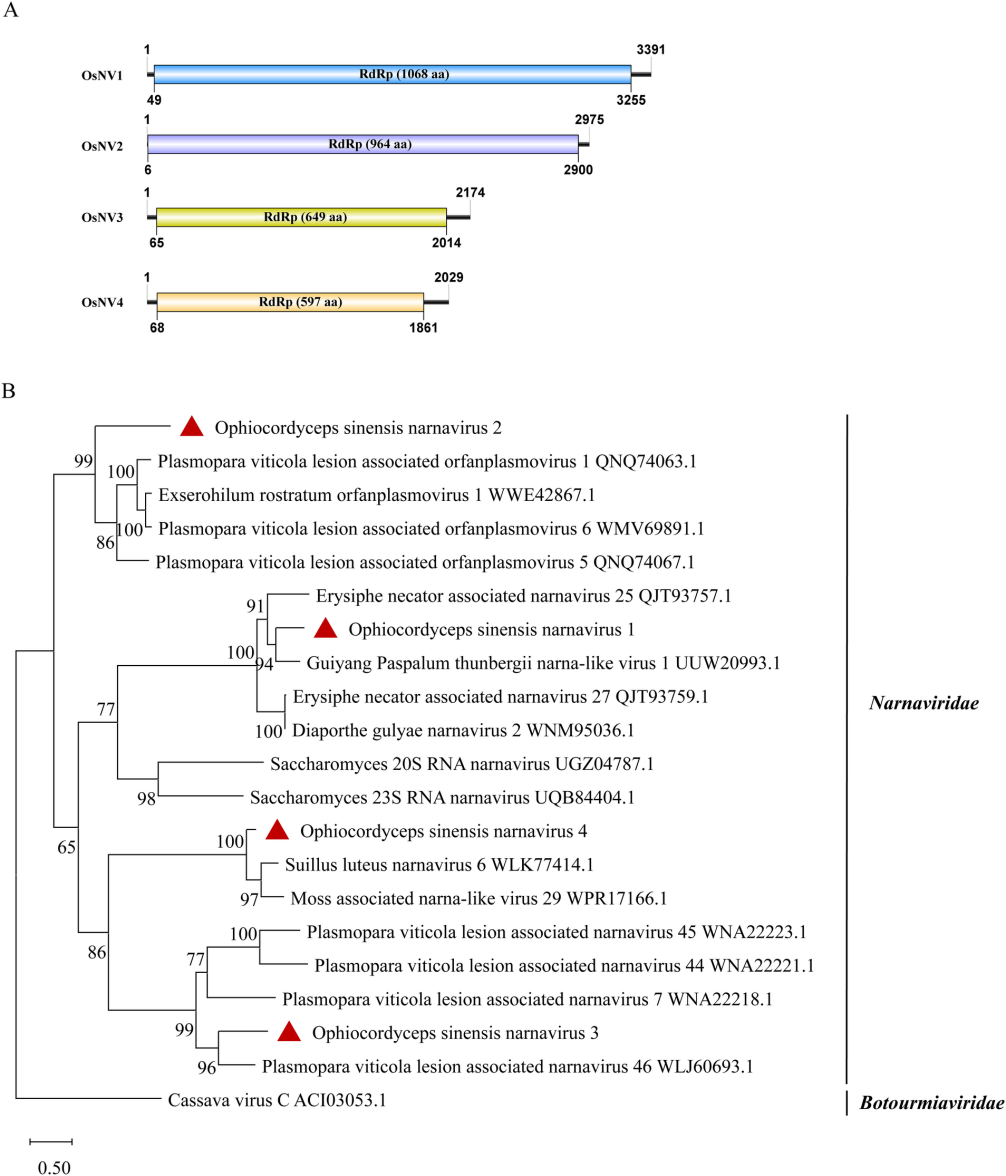
Genome organization and phylogenetic analysis of OsNV1–4. (A) Genomic structures of OsNV1–4. ORFs are shown as boxes. (B) Evolutionary relationships of the viruses in the *Narnaviridae* family. RdRp amino acid sequences from different genera of *Narnaviridae* were aligned to derive a phylogenetic tree using the maximum likelihood method with the LG + I + G substitution model. *Botourmiaviridae* is set as the outgroup. Bootstrap values (%) of 1,000 replicates that are greater than 50% are labeled on the branches. The viruses OsNV1–4 are marked with a red triangle.

According to the ICTV classification, the *Narnaviridae* has only one genus, the *narnavirus*. A typical narnavirus lacks a capsid and encodes a single RdRp protein. We have identified four new narnaviruses in *O. sinensis*. All the viruses exhibit a single large ORF encoding an RdRp. They were designated as Ophiocordyceps sinensis narnavirus 1, 2, 3 and 4 (OsNV1, OsNV2, OsNV3, OsNV4). OsNV1 is comprised of 3,391 nt and encodes a 1,068-aa RdRp (Figure 5A). BLASTx analysis revealed that the RdRp of OsNV1 shares 54.1% identity with that of Guiyang Paspalum thunbergii narna-like virus 1 (query coverage: 93%; *E*-value: 0; and accession: UUW20993.1). OsNV2 has a full length of 2,975 nt and encodes a 964-aa RdRp protein showing the highest sequence identity of 39.0% with the RdRp of Downy mildew lesion associated orfanplasmovirus 1 (query coverage: 71%; *E*-value: 3e^−133^; and accession: QNQ74063.1). Interestingly, ambigrammatic narnaviruses can harbor an additional, reverse frame ORF (rORF) overlapping the RdRp ORF. The rORF spans almost the entire length of the viral genome (Retallack et al., 2021; Zhang et al., 2023). Instead of a large rORF, OsNV2 presents two smaller reverse-frame ORFs with sizes of 78-aa and 89-aa. OsNV3 and OsNV4, with full lengths of 2,174 nt and 2029 nt, respectively. OsNV3 encodes a 649-aa RdRp and shares 45.6% identity with the RdRp of Downy mildew lesion associated splipalmivirus 46 (query coverage: 88%; E-value: 0; and accession: WLJ60693.1), while OsNV4’s 597-aa RdRp shows 63.5% identity with Suillus luteus narnavirus 6 (query coverage: 86%; E-value: 0; and accession: WLK77414.1). The phylogenetic analysis supported the classification of OsNV1–4 as new species within the family *Narnaviridae* (Figure 5B).

### 3.8 *Partitiviridae*-related sequences

**Figure 6:**
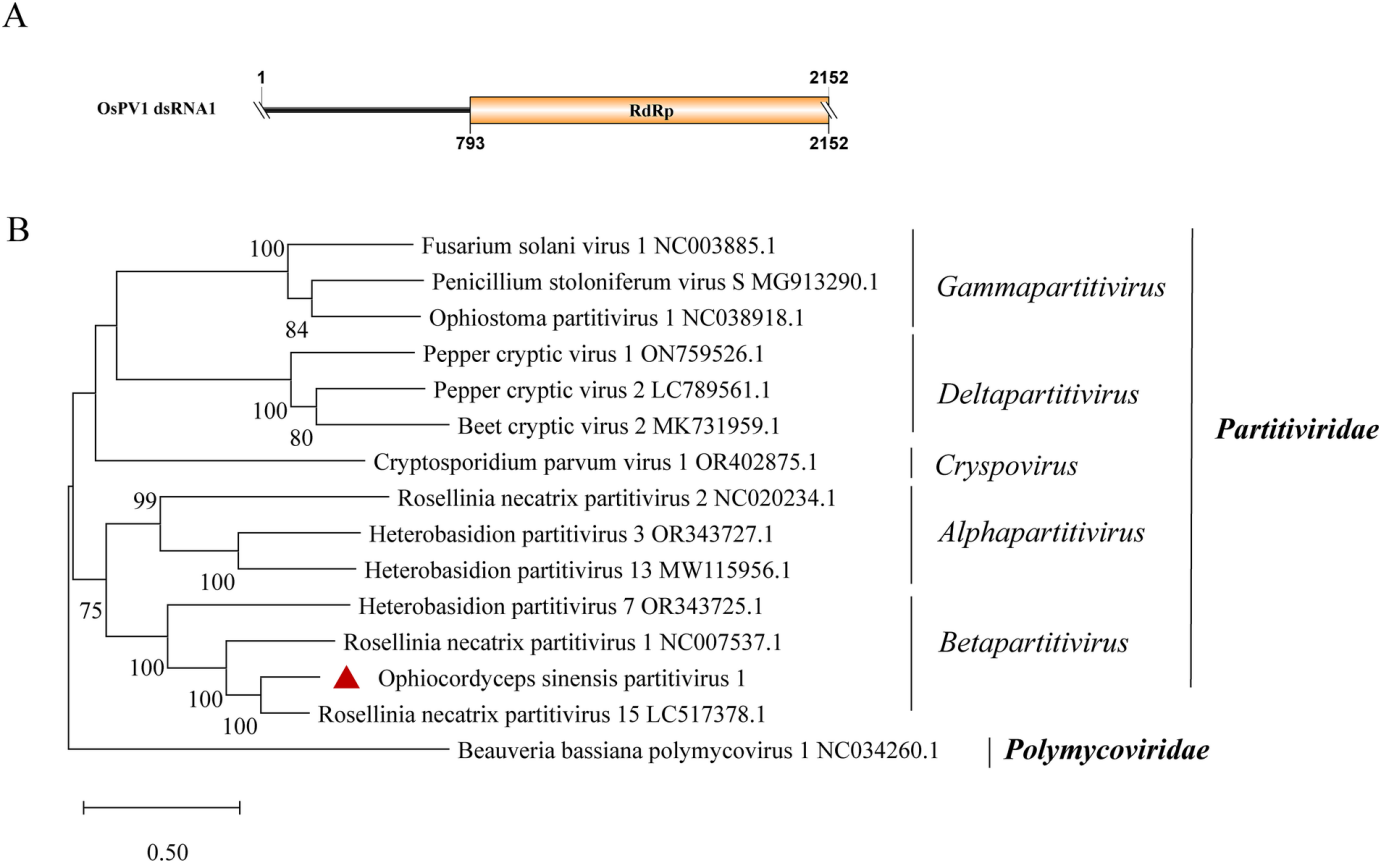
Genome organization and phylogenetic analysis of OsPV1. (A) Genomic structure of OsPV1. ORF is shown as a box. The sequence of the genome is incomplete. (B) Evolutionary relationships of the viruses in the *Partitiviridae* family. RdRp nucleotide sequences from different genera of *Partitiviridae* were aligned to derive a phylogenetic tree using the maximum likelihood method with the GTR + I + G substitution model. *Polymycoviridae* is set as the outgroup. Bootstrap values (%) of 1,000 replicates that are greater than 50% are labeled on the branches. The virus OsPV1 is marked with a red triangle.

A new dsRNA partitivirus, named Ophiocordyceps sinensis partitivirus 1 (OsPV1), was discovered in sample SRR5428527. This marks the first report of a partitivirus in *O. sinensis*. Typically, partitiviruses have two dsRNA genomic segments, one of which encodes an RdRp and the other encodes a CP (Zhu et al., 2024). However, only one contig of 2,152 nt was found in OsPV1 (Figure 6A). It encodes an incomplete RdRp sequence with 76.9% identity to the RdRp in Rosellinia necatrix partitivirus 15 (query coverage: 99%; *E*-value: 0; and accession: BBU59838.1). The segment encoding the CP was not detected in OsPV1. Based on phylogenetic analysis, OsPV1 showed a close evolutionary relationship with members of the genus *Betapartitivirus* (Figure 6B).

### 3.9 Vivivirus-related sequences

**Figure 7:**
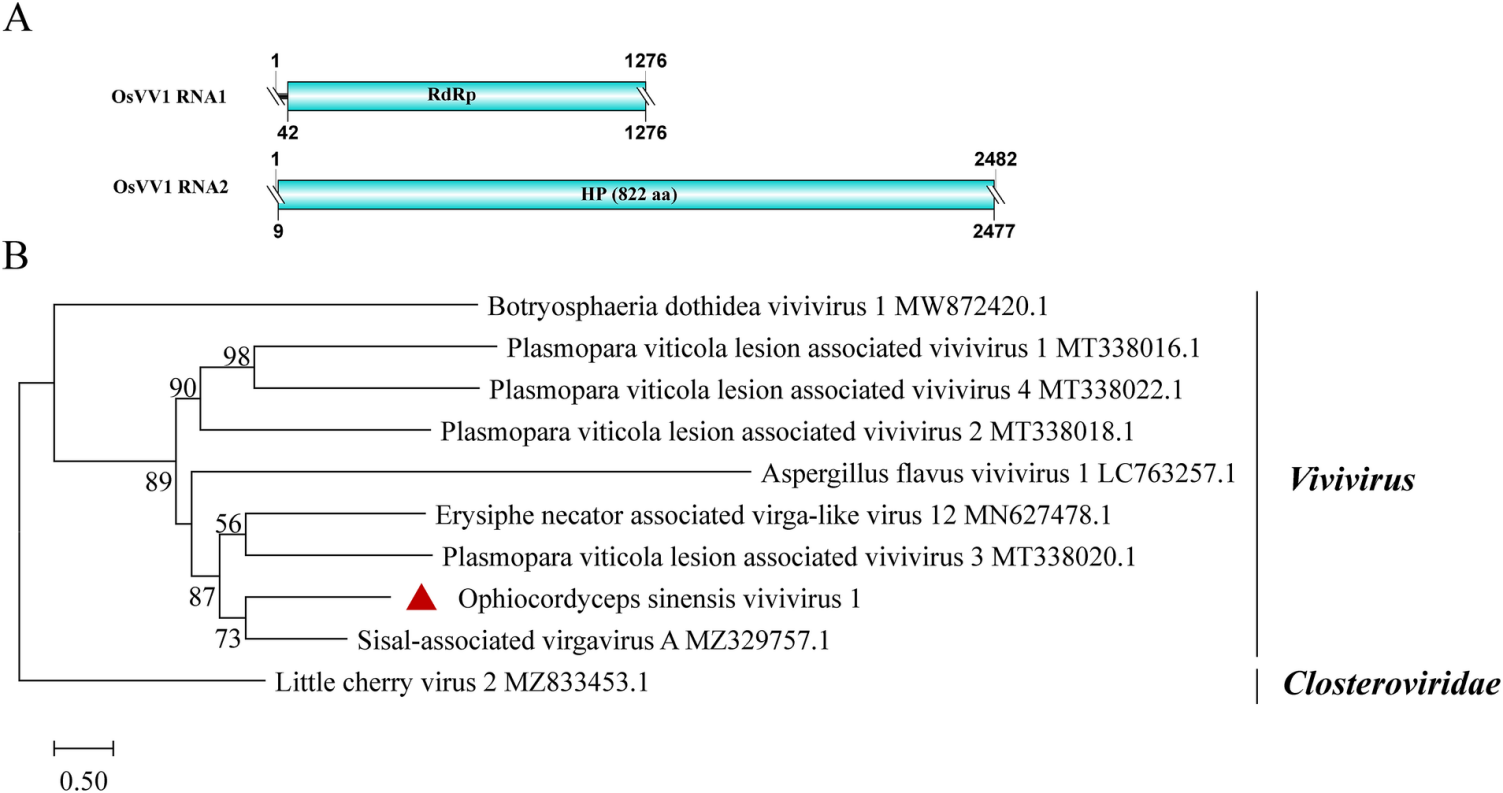
Genome organization and phylogenetic analysis of OsVV1. (A) Genome structures of OsVV1. ORFs are shown as boxes. The sequences of the genomes are all incomplete. (B) Evolutionary relationships of the viruses in the viviviruses. RdRp nucleotide sequences from different viviviruses were aligned to derive a phylogenetic tree using the maximum likelihood method with the GTR + I + G substitution model. *Closteroviridae* is set as the outgroup. Bootstrap values (%) of 1,000 replicates that are greater than 50% are labeled on the branches. The virus OsVV1 is marked with a red triangle.

A new vivivirus, named Ophiocordyceps sinensis vivivirus 1 (OsVV1), was identified in this study. It is the first time for vivivirus identified among entomopathogenic fungi. Viviviruses, related phylogenetically to plant virgaviruses, have been discovered in phytopathogenic fungi. Vivivirus typically possesses two to three (+) ssRNA genomic segments, one of which expresses methyltransferase (Mtr) and helicase (Hel), the other expresses RdRp and sometimes expresses another Mtr (Chiapello et al., 2020; Degola et al., 2021; Kondo et al., 2022). Sample SRR8258357 contained two contigs, with lengths of 1,276 nt and 2,482 nt, respectively (Figure 7A). BLASTx analysis revealed that the shorter contig had 52.4% identity to RdRp in Sisal-associated virgavirus A (query coverage: 93%; E-value: 1e^−122^; and accession: QYJ09848.1). Phylogenetic analysis showed a close relationship between OsVV1 and other viviviruses, supporting its identification (Figure 7B). The other contig showed 27.9% identity to a hypothetical protein of Aspergillus flavus vivivirus 1 (query coverage: 56%; *E*-value: 6e^−20^; and accession: BED98313.1). In addition, the structural domain encoding Mtr was not found.

### 3.10 *Deltaflexiviridae*-related sequences

**Figure 8:**
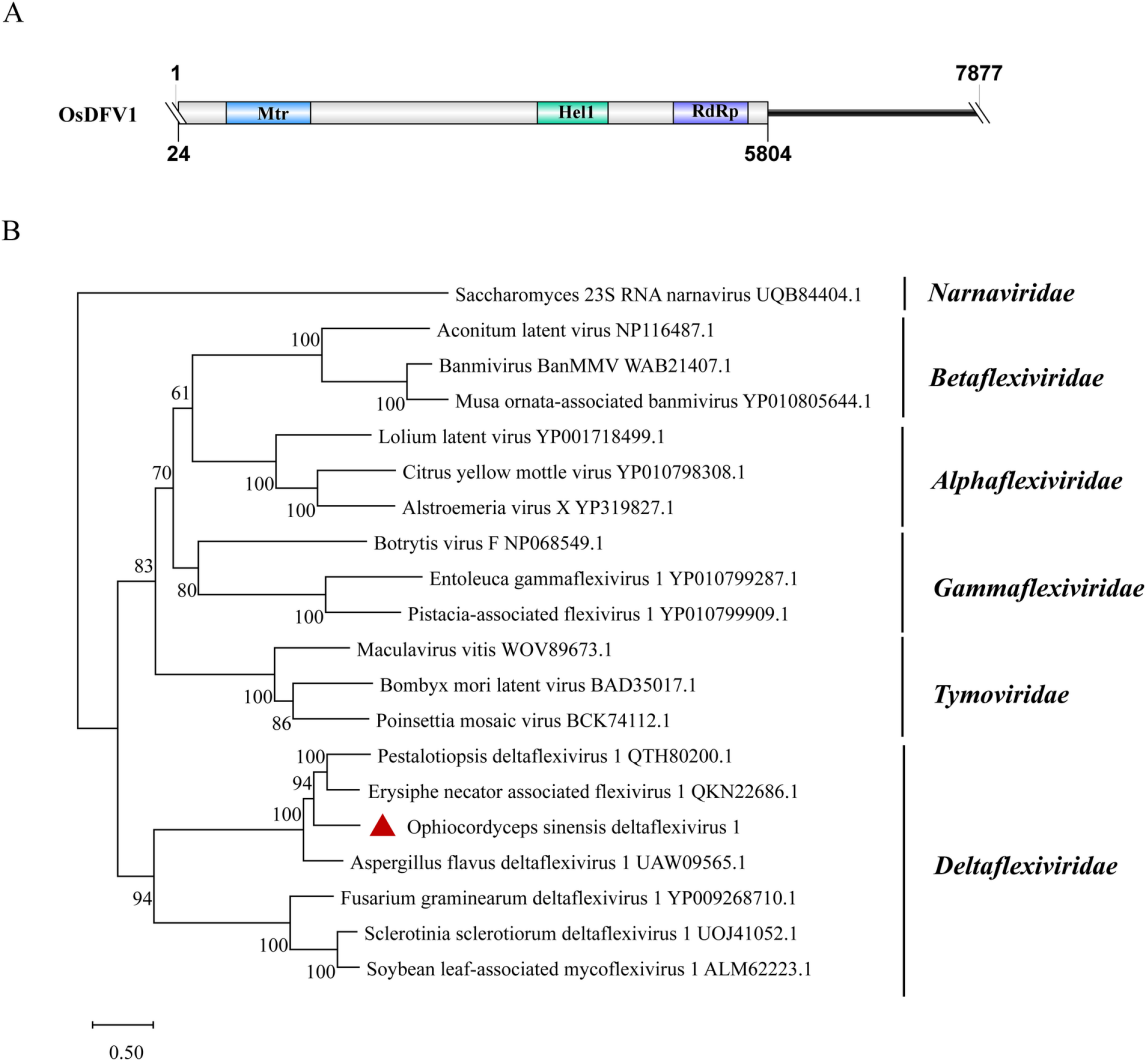
Genome organization and phylogenetic analysis of OsDFV1. (A) Genome structures of OsDFV1. ORFs are shown as boxes. The sequence of the genome is incomplete. (B) Evolutionary relationships of the viruses in the *Tymovirales*. RdRp amino acid sequences from different families of *Tymovirales* were aligned to derive a phylogenetic tree using the maximum likelihood method with the LG + I + G substitution model. *Narnaviridae* is set as the outgroup. Bootstrap values (%) of 1,000 replicates that are greater than 50% are labeled on the branches. The virus OsDFV1 is marked with a red triangle.

A contig of 7,877 nt from samples SRR12952889 and SRR8258357 showed 56.9% identity to the RdRp of Erysiphe necator-associated flexivirus 1 (EnaFV1) (query coverage: 69%; *E*-value: 0; and accession: QKN22686.1). This contig contains a large ORF encoding 1926 aa, which was predicted to present three structural domains: Mtr, Hel1 and RdRP (Figure 8A). Flexivirues can be classified into five families: *Alphaflexiviridae*, *Betaflexiviridae*, *Deltaflexiviridae*, *Gammaflexiviridae* and *Tymoviridae* (Ye et al., 2023). Based on phylogenetic analysis, this novel flexivirus exhibited a close evolutionary relationship with members in the *Deltaflexiviridae* family (Figure 8B) and thus was designated as Ophiocordyceps sinensis deltaflexivirus 1 (OsDFV1).

### 3.11 Divergent viruses were detected in the data from Chinese cordyceps at different developmental stages

Samples of three distinct *O. sinensis* strains were analyzed across their various developmental stages. It is shown that distinct viral species and loads were observed in the data from the same strain at various points during the fungal lifecycle (Table 3 and Supplementary Table S5).

**Table 3:** Summary of Chinese cordyceps mycovirus genomes at different developmental stages identified from transcriptomic datasets.

		Tissue	OsOMV1	OsOVA	OsMV1	OsMV2	OsMV3	OsMV4	OsNV1	OsNV2	OsNV3	OsNV4	OsPV1	OsVV1	OsDFV1	Total number
PRJNA 507459 (Li et al., 2019)	SRR8258340	Hyphae	+ (37)	+ (38)	+ (20)	+ (19)	+ (395)			+ (35)	+ (1057)	+ (10588)				8
	SRR8258343	Sclerotium	+ (205)	+ (84)			+ (46)		+ (8820)							4
	SRR8258346	Primordium		+ (184)					+ (98)			+ (14)			+ (1143)	4
	SRR8258349	Young fruitbody	+ (15)	+ (144)					+ (32146)	+ (21)	+ (487)	+ (4575)			+ (106)	7
	SRR8258352	Developed fruitbody	+ (109)	+ (293)			+ (167)		+ (23659)		+ (21)	+ (56)			+ (358)	7
	SRR8258357	Mature fruitbody	+ (27)	+ (110)				+ (13)	+ (21)			+ (22)		+ (29)	+ (1725)	7
PRJNA 673413 Stain SICC 5.02 (Zhang et al., 2021)	SRR12952889	Mycoparasite complex	+ (14)	+ (69)			+ (134)		+ (49)						+ (16376)	5
	SRR12952892	Sclerotium	+ (7)							+ (19)						2
	SRR12952895	Fruitbody														0
PRJNA 625214 Stain IOZ07 (Li et al., 2020)	SRR11548640	Cultured mycelia				+ (934)	+ (2113)									2
	SRR11547913	Blastospores in proliferative stage	+ (7)			+ (102)			+ (38157)							3
	SRR11547906	Blastospores in stationary stage	+ (72)			+ (95)	+ (16)		+ (7096)	+ (315)						5
	SRR11547905	Prehyphae	+ (38)													1
	SRR11547910	Hyphae	+ (58)						+ (13486)							2
PRJNA 600609 Stain IOZ07 (Zhao et al., 2020)	SRR10878126	Sclerotium	+ (355)	+ (700)						+ (43)						3
	SRR10878121	Primordium	+ (211)							+ (66)						2

OsOMV1, Ophiocordyceps sinensis ormycovirus 1; OsOVA, Ophiocordyceps ourmiavirus A; OsMV, Ophiocordyceps sinensis mitovirus; OsNV, Ophiocordyceps sinensis narnavirus; OsPV1, Ophiocordyceps sinensis partitivirus 1; OsVV1, Ophiocordyceps sinensis vivivirus 1; OsDFV1, Ophiocordyceps sinensis deltaflexivirus 1. The ‘+’ indicates the presence of mycovirus in the sample and the number in parentheses represents the number of reads for each mycovirus in the sample.

In PRJNA507459 (Supplementary Table S6), Chinese cordyceps was classified into six distinct developmental stages. The hyphae sample exhibited the highest viral count. Samples from young fruitbody, developed fruitbody and mature fruitbody, were detected with an equal number of viruses, hosted different viral species. In PRJNA673413 (Supplementary Table S7), Chinese cordyceps was divided into three developmental stages. The highest number of viruses was detected in the larva-fungus complex sample, suggesting a possible influence of the interaction between fungus and host on viral diversity or load. Interestingly, none of the 13 viruses mentioned above was identified in the fruitbody sample, highlighting the possibility of stage-specific viral resistance or clearance mechanisms in this strain. PRJNA625214 (Supplementary Table S8) and PRJNA600609 (Supplementary Table S9) represent the same strain with a total of seven distinct developmental stages. Viruses were detected in all the stages, with the highest virus counts being in the blastospores in stationary stage, containing five distinct species. Notably, OsOMV1 is consistently present in six developmental stages examined. Overall, the number of viruses carried did not favor any specific developmental stage.

## 4 Discussion

The study has isolated and sequenced dsRNA in *O. sinensis* strains. By combining the analysis of publicly available transcriptome data from the SRA, we provide a comprehensive report on the diversity of mycovirus within *O. sinensis*. We identified 13 distinct mycoviruses, obtaining the full genomes of eight through RACE, while assembling the remaining five from public data, albeit with incomplete sequences. Most of these viruses possess a (+) ssRNA genome, except for one dsRNA virus. These mycoviruses span five families (*Partitiviridae*, *Mitoviridae*, *Narnaviridae*, *Botourmiaviridae*, *Deltaflexiviridae*) as well as two unclassified lineages (Ormycovirus and Vivivirus). Notably, the *Mitoviridae* and *Narnaviridae* families were the most prevalent (Table 4), suggesting their significant ecological or evolutionary roles in *O. sinensis*.

**Table 4:** Summary of mycoviruses in this research.

Virus name	Family	Genus	Group	Data Source	Complete^a^	Segment number	Length (nt)	Nucleotide accession
Ophiocordyceps sinensis partitivirus 1 (OsPV1)	*Partitiviridae*	*Betapartitivirus*	dsRNA	SRA	N	1	2152	BK068004
Ophiocordyceps sinensis mitovirus 1 (OsMV1)	*Mitoviridae*	*Unuamitovirus*	+ssRNA	SRA	Y	1	2386	MK279484.1
Ophiocordyceps sinensis mitovirus 2 (OsMV2)		*Unuamitovirus*		Transcriptome	Y	1	2439	MK279485.1
Ophiocordyceps sinensis mitovirus 3 (OsMV3)		*Unuamitovirus*		Transcriptome	Y	1	2600	PP623133
Ophiocordyceps sinensis mitovirus 4 (OsMV4)		*Unuamitovirus*		SRA	N	1	2501	BK068008
Ophiocordyceps sinensis narnavirus 1 (OsNV1)	*Narnaviridae*	*Narnavirus*		Transcriptome	Y	1	3391	PP623134
Ophiocordyceps sinensis narnavirus 2 (OsNV2)		*Narnavirus*		Transcriptome	Y	1	2975	PP623135
Ophiocordyceps sinensis narnavirus 3 (OsNV3)		*Narnavirus*		Transcriptome	Y	1	2174	PP623136
Ophiocordyceps sinensis narnavirus 4 (OsNV4)		*Narnavirus*		Transcriptome	Y	1	2029	PP623137
Ophiocordyceps sinensis ormycovirus 1 (OsOMV1)	Ormycovirus			Transcriptome	Y	1	2506	PP623130
						2	2156	PP623131
Ophiocordyceps ourmiavirus A (OsOVA)	*Botourmiaviridae*	*Gammascleroulivirus*		Transcriptome	Y	1	2967	PP623132
Ophiocordyceps sinensis vivivirus 1 (OsVV1)	Vivivirus			SRA	N	1	1276	BK068005
					N	2	2482	BK068006
Ophiocordyceps sinensis deltaflexivirus 1 (OsDFV1)	*Deltaflexiviridae*	*Deltaflexivirus*		SRA	N	1	7877	BK068007

^a^ Y and N represent complete and incomplete sequences, respectively.

*Mitoviridae* viruses, typically localizing within mitochondria, provide stability and resistance against the host’s detoxification, and may evade host antiviral RNA silencing mechanisms (Shahi et al., 2019). Currently, the *Mitoviridae* family is divided into four distinct genera. Our phylogenetic analysis suggests OsMV1-4 all belong to the genus *Unuamitovirus*. The *Narnaviridae* family exhibits remarkable diversity in terms of genome structure and host range (Hillman and Cai, 2013). OsNV1–4, detected in this study, are typical of narnaviruses, possessing an unsegmented, linear (+) ssRNA genome without rORFs. The simplicity of these viral genome structures could indicate a streamlined replication strategy that may confer evolutionary advantages. A thorough search for ORFans led to the discovery of a unique group of highly differentiated RNA arboviruses, designated as ormycovirus. SbOMV1 is currently the only ormycovirus collected from a corresponding live biological sample. OsOMV1 was identified earlier using publicly accessible data (Forgia et al., 2022). We detected OsOMV1 in a dsRNA sample from the IOZ strain, offering compelling evidence for the existence of ormycovirus. Notably, this virus was abundantly present in the SRA samples analyzed, highlighting its potential significance in the Chinese cordyceps lifecycle.

Coinfection of mycoviruses is a common phenomenon. The likelihood of coinfection in nature is much greater than that of single mycovirus infections (Thapa and Roossinck, 2019). For instance, *Sclerotinia sclerotiorum* SX276 was infected by nine mycoviruses from different evolutionary lineages, including seven (+) ssRNA viruses, one dsRNA virus, and one negative-sense ssRNA virus. This coinfection resulted in an altered growth rate, abnormal colony morphology, and reduced pathogenicity, characteristics typical of a hypovirulent strain (Mu et al., 2021). *B. bassiana* isolates infected with Beauveria bassiana polymycovirus 1 (BbPmV-1) and Beauveria bassiana non-segmented virus 1 (BbNV-1) exhibited increased growth rate, biomass, and hypervirulence towards *Galleria mellonella* larvae (Kotta-Loizou and Coutts, 2017). According to our observation, *O. sinensis* infecting its host larvae differs from the infection patterns of entomopathogenic fungi, such as *B. bassiana* and *Metarhizium robertsii*. The fungus can evade host immune responses and sustain a prolonged infection (5 to 12 months) without inducing disease symptoms on the host body surface (Meng et al., 2021; Wu et al., 2022b). Among the IOZ strains, IOZ14 and IOZ29 harbored eight viruses, while the other strains contained seven. Except for SRR13286702 and SRR5446809, all other SRA samples displayed coinfections. Notably, data from the larva-fungus complex showed the highest viral count compared to data from other tissues in PRJNA673413. The prevalence of coinfection within *O. sinensis* suggests a complex viral ecology that may influence the fungal ability to infect and persist in the host over the long-term infection. According to our observations on the mycelia, no visible morphological differences among the six IOZ strains were noted, except that the colonies of strain IOZ31 were darker in color. Due to the diversity of mycoviruses present in the *O. sinensis* strains, it was challenging to determine their specific effects on the biological phenotypes of these strains. Isolating stains with a single mycovirus would help us unravel these complex mechanisms.

Chinese cordyceps has attracted significant attention due to its limited distribution range, mysterious lifecycle, ecological importance, and developmental biology. Based on the process of artificial cultivation of *O. sinensis*, the development of its fruiting body can be divided into different stages. During the analysis of the SRA samples, it was discovered that the data from the same strain showed various amounts and types of mycoviruses at different developmental stages (Table 3), suggesting a dynamic shift in viral composition during its development. However, the viral distribution did not show a clear preference. This might be because of their geographically disparate habits or the limited number of analyzed samples. OsOMV1 was identified in SRR10878121 sample but not in the SRR8258346 sample. Both samples were collected at the primordium stage. The virus could be consistently detected in all other developmental stage samples belonging to the same strain as SRR8258346. Viruses that ‘disappear’ within a strain at a certain stage were probably due to their low abundances and insufficient sequencing depth. Investigating the mycoviral abundance and distribution within more *O. sinensis* strains during the entire lifecycle may reveal virus-host dynamics and their ecological consequences.

## 5 Conclusion

In summary, this study not only maps the mycoviral landscape of *O. sinensis* but also opens new avenues for research into the ecological and evolutionary implications of mycoviruses in fungi. The insights gained here pave the way for studies exploring the intricate relationships between mycoviruses and their hosts, with potential applications in fungal biology, ecology, and biotechnology.

## Data Availability

The viral genomes characterized for this study can be found in the NCBI with the accession numbers listed in Table 4 for each viral genome segment. The IOZ strains transcriptome raw reads have been deposited in the SRA with accession number PRJNA1169795.

## Footnotes

^1^ https://www.ncbi.nlm.nih.gov/sra

^2^ https://www.ncbi.nlm.nih.gov/orffinder/

^3^ https://www.ncbi.nlm.nih.gov/Structure/cdd/wrpsb.cgi

## Supplementary material

Supplementary Table S1 Primer pairs used to confirm viral sequences in IOZ strains.

Supplementary Table S2 The terminal primer of the eight viruses in IOZ strains with RACE.

Supplementary Table S3 Detailed information on contigs obtained from 13 different samples by analyzing the SRA database.

Supplementary Table S4 Coverage (% genome length) of each mycovirus identified in the public transcriptome of different O. sinensis strains.

Supplementary Table S5 Coverage (% genome length) of each mycovirus identified in the public transcriptome of Chinese cordyceps at different developmental stages.

Supplementary Table S6 Detailed information on contigs obtained from 6 different samples by analyzing the PRJNA507459.

Supplementary Table S7 Detailed information on contigs obtained from 3 different samples by analyzing the PRJNA673413.

Supplementary Table S8 Detailed information on contigs obtained from 5 different samples by analyzing the PRJNA625214.

Supplementary Table S9 Detailed information on contigs obtained from 2 different samples by analyzing the PRJNA600609.
