# Supplementary material for: Unveiling mycoviral diversity in Ophiocordyceps sinensis through transcriptome analyses
Source: Front Microbiol. 2024 Nov 25;15:1493365. doi: 10.3389/fmicb.2024.1493365 (PMC11625762; doi:10.3389/fmicb.2024.1493365)
Supplement: Supplementary Table S1 — Primer pairs used to confirm viral sequences in IOZ strains. [file Table_1.docx]

Table S1 Primer pairs used to confirm viral sequences in IOZ strains.

| Primer name | Primer sequence (5′-3′) | Product length (bp) |
| --- | --- | --- |
| OsOMV1_RNA1_testF | AGGCTGTGGGCTTCAATACC |  |
| OsOMV1_RNA1_testR | TTCGCTGCCTTCTGTAGGTG | 673 |
| OsOMV1_RNA2_testF | AGAAGAAATGGGGACGGTCG |  |
| OsOMV1_RNA2_testR | GCGAGAAGTTCGGTCAAGGA | 603 |
| OsOVA_testF | AAGTGCGTAGGGGAAACGCA |  |
| OsOVA_testR | CATTGCGCAGCAGAGAATCC | 568 |
| OsMV2_testF | AAGGTCCAAATGGTCATGCT |  |
| OsMV2_testR | AACGATCCATAGGCTCCCATT | 589 |
| OsMV3_testF | GTAGTCCGTATCGCAGCACA |  |
| OsMV3_testR | GGGCGAGATTTCAAGCGAAC | 598 |
| OsNV1_testF | AGGGCGAAAGAATGGATGGG |  |
| OsNV1_testR | CCACGGAGCGATATCAGGAC | 563 |
| OsNV2_testF | CTTTGGGTTGCGGTCAAGTG |  |
| OsNV2_testR | GAAGTCGGATCGCCCATCAT | 602 |
| OsNV3_testF | GGACAATGCTTCCGGTCAGA |  |
| OsNV3_testR | CCAGTCAGCGTGTACCCTTT | 665 |
| OsNV4_testF | TTCCCAGACTCACCCCAGAT |  |
| OsNV4_testR | ACAATGAACGGGTTGGCGTA | 1013 |
| Osactin_F | GCAACTACATGAACCGCGAC |  |
| Osactin_R | CCACATCTGGTTGTACCCGT | 755 |
