# Supplementary material for: Unveiling mycoviral diversity in Ophiocordyceps sinensis through transcriptome analyses
Source: Front Microbiol. 2024 Nov 25;15:1493365. doi: 10.3389/fmicb.2024.1493365 (PMC11625762; doi:10.3389/fmicb.2024.1493365)
Supplement: Supplementary Table S2 — The terminal primer of the eight viruses in IOZ strains with RACE. [file Table_2.docx]

Table S2 The terminal primer of the eight viruses in IOZ strains with RACE.

| Primer name | Primer sequence (5′-3′) |
| --- | --- |
| RACE-oligo | GCATTGCATCATGATCGATCGAATTCTT  TAGTGAGGGTTAATTGCC-(NH_2_) |
| RACE1 | GGCAATTAACCCTCACTAAAG |
| RACE2 | TCACTAAAGAATTCGATCGATC |
| RACE3 | CGATCGATCATGATGCAATGC |
| OsOMV1_RNA1_F1 | CTGCATACGTTGCCAATAAG |
| OsOMV1_RNA1_F2 | CATGATTCCTGGTCTTTTGC |
| OsOMV1_RNA1_R1 | AGCTCGGCTTTATAACAACA |
| OsOMV1_RNA1_R2 | GATAAGATGCAGTCCCATGT |
| OsOMV1_RNA2_F1 | CTTCGATGGTATCCACTGTT |
| OsOMV1_RNA2_F2 | ACGCGAAACAGTTAAGAAAC |
| OsOMV1_RNA2_R1 | AGACGTTTCAAAACCTAGCA |
| OsOMV1_RNA2_R2 | TGACTGTAGGAGTAGGGAAG |
| OsOVA_F1 | TATGTGACTCCAGGCTTACT |
| OsOVA_F2 | TTACACAGGATGGGTCTTCT |
| OsOVA_R1 | GATAAGCTTGTGCAAAGGAC |
| OsOVA_R2 | TCTTCATTGTCCTCCCCTAT |
| OsMV2_F1 | TTCGAAAGTGATGAAGAGGA |
| OsMV2_F2 | CCCAGTACCTGTCTAATTGA |
| OsMV2_R1 | TTACGGGTTGTGTGATTGAA |
| OsMV2_R2 | CTTTGTCACAACGTGAGGTG |
| OsMV3_F1 | CGAAGCTCGATTAGAGAGAG |
| OsMV3_F2 | CTGTTTTGAGAGCGAGAAAC |
| OsMV3_R1 | CTCCGAGGTGATTGTTGTTA |
| OsMV3_R2 | TTGGGTAATCCGACTTTTGT |
| OsNV1_F1 | ACATCGTGGTCTTTGTTACT |
| OsNV1_F2 | GTTCAAAACCGCTCTATTCG |
| OsNV1_R1 | CAACGATCTTCTTGACCCAA |
| OsNV1_R2 | TTAAGATGGAGAACCCCCTT |
| OsNV2_F1 | CCTTTAAGAAAGGTGCCTCA |
| OsNV2_F2 | CTGATCATGTTGAGCGTACC |
| OsNV2_R1 | TACGTACTCTCTGGAAACCA |
| OsNV2_R2 | GTTGCCTGCAAACATAGGTA |
| OsNV3_F1 | CCGATGAAGGGGTCATTATC |
| OsNV3_F2 | ACATTGAGACTCTTCGGACT |
| OsNV3_R1 | TTACCGATGTCTGTTGAGGA |
| OsNV3_R2 | AATTGCCGATTCCCTCATTT |
| OsNV4_F1 | TACGCCAACCCGTTCATTGT |
| OsNV4_F2 | GGGTGCCTTATGGGTGATCC |
| OsNV4_R1 | ATCTGGGGTGAGTCTGGGAA |
| OsNV4_R2 | GTCACGTGTGACCGTTTTCG |

Two pairs of nested primers, F1/R1 and F2/R2. F1/F2 and R1/R2 were designed for 3'- and 5'-terminal sequence cloning by nested PCR. The specific primer F1/R1, which is distant from the end of the sequence, was combined with RACE2 for PCR amplification. The first PCR product was diluted 10-fold and used as a template for the second PCR. The second round of PCR was amplified using specific primers F2/R2, which are closer to the end of the sequence, in combination with RACE3.
