# Supplementary material for: Unveiling mycoviral diversity in Ophiocordyceps sinensis through transcriptome analyses
Source: Front Microbiol. 2024 Nov 25;15:1493365. doi: 10.3389/fmicb.2024.1493365 (PMC11625762; doi:10.3389/fmicb.2024.1493365)
Supplement: Supplementary Table S3 — Detailed information on contigs obtained from 13 different samples by analyzing the SRA database. [file Table_3.docx]

Table S3 Detailed information on contigs obtained from 13 different samples by analyzing the SRA database.

| SRA ID | contig | protein description | Length (nt) | Ident (%) | Name of putative virus |
| --- | --- | --- | --- | --- | --- |
| SRR11548640 | k141_726 | AZT88623.1 RNA-dependent RNA polymerase [Ophiocordyceps sinensis mitovirus 1] | 2565 | 50.5 | Ophiocordyceps sinensis mitovirus 3 |
|  | k141_187 | AZT88624.1 RNA-dependent RNA polymerase [Ophiocordyceps sinensis mitovirus 2] | 2396 | 97.9 | Ophiocordyceps sinensis mitovirus 2 |
| SRR12952889 | k141_1472 | QKN22686.1 RNA-dependent RNA polymerase [Erysiphe necator associated flexivirus 1] | 7850 | 56.9 | Ophiocordyceps sinensis deltaflexivirus 1 |
|  | k141_1605 | AHF48631.1 RNA-dependent RNA polymerase [Sclerotinia sclerotiorum mitovirus 15] | 1744 | 53.6 | Ophiocordyceps sinensis mitovirus 3 |
|  | k141_2172 | UUW20993.1 MAG: RNA-dependent RNA polymerase [Guiyang Paspalum thunbergii narna-like virus 1] | 923 | 48.1 | Ophiocordyceps sinensis narnavirus 1 |
|  | k141_3419 | UUW20993.1 MAG: RNA-dependent RNA polymerase [Guiyang Paspalum thunbergii narna-like virus 1] | 898 | 65.8 | Ophiocordyceps sinensis narnavirus 1 |
|  | k141_2958 | UUW20993.1 MAG: RNA-dependent RNA polymerase [Guiyang Paspalum thunbergii narna-like virus 1] | 856 | 52.1 | Ophiocordyceps sinensis narnavirus 1 |
|  | k141_1653 | USW07202.1 putative RNA-dependent RNA polymerase [Plasmopara viticola lesion-associated ormycovirus 3] | 760 | 46.6 | Ophiocordyceps sinensis ormycovirus 1 |
|  | k141_2125 | USW07204.1 putative RNA-dependent RNA polymerase [Erysiphe lesion-associated ormycovirus 3] | 733 | 44.8 | Ophiocordyceps sinensis ormycovirus 1 |
|  | k141_276 | UYL95443.1 MAG: RNA-dependent RNA polymerase [Hulunbuir Botou tick virus 5] | 642 | 51.9 | Ophiocordyceps ourmiavirus A |
|  | k141_1296 | AZT88623.1 RNA-dependent RNA polymerase [Ophiocordyceps sinensis mitovirus 1] | 608 | 46.1 | Ophiocordyceps sinensis mitovirus 3 |
|  | k141_1007 | USW07207.1 putative RNA-dependent RNA polymerase [Erysiphe lesion-associated ormycovirus 2] | 530 | 50.9 | Ophiocordyceps sinensis ormycovirus 1 |
|  | k141_3613 | UPO93688.1 coat protein [Metarhizium brunneum bipartite mycovirus 1] | 521 | 76.3 |  |
|  | k141_1703 | USW07207.1 putative RNA-dependent RNA polymerase [Erysiphe lesion-associated ormycovirus 2] | 478 | 41.6 | Ophiocordyceps sinensis ormycovirus 1 |
|  | k141_3924 | USW07212.1 hypothetical protein [Erysiphe lesion-associated ormycovirus 2] | 398 | 42.1 | Ophiocordyceps sinensis ormycovirus 1 |
|  | k141_2872 | ELU41375.1 pneumovirus matrix domain-containing protein [Rhizoctonia solani AG-1 IA] | 348 | 77.8 |  |
|  | k141_495 | USW07203.1 hypothetical protein [Plasmopara viticola lesion-associated ormycovirus 3] | 328 | 42.1 | Ophiocordyceps sinensis ormycovirus 1 |
| SRR13286702 | k141_102 | USW07212.1 hypothetical protein [Erysiphe lesion-associated ormycovirus 2] | 415 | 39.1 | Ophiocordyceps sinensis ormycovirus 1 |
|  | k141_438 | ANF07154.1 nucleocapsid protein, partial [Canine parainfluenza virus] | 340 | 98.2 |  |
|  | k141_58 | EPJ19128.1 retrovirus-related Pol polyLINE-1 domain protein, partial [Chlamydia psittaci 02DC22] | 311 | 46.4 |  |
| SRR21290686 | k141_1931 | AZT88623.1 RNA-dependent RNA polymerase [Ophiocordyceps sinensis mitovirus 1] | 2500 | 50.3 | Ophiocordyceps sinensis mitovirus 3 |
|  | k141_2579 | USW07207.1 putative RNA-dependent RNA polymerase [Erysiphe lesion-associated ormycovirus 2] | 2421 | 46 | Ophiocordyceps sinensis ormycovirus 1 |
|  | k141_1764 | USW07212.1 hypothetical protein [Erysiphe lesion-associated ormycovirus 2] | 2115 | 34.1 |  |
|  | k141_1809 | AZT88624.1 RNA-dependent RNA polymerase [Ophiocordyceps sinensis mitovirus 2] | 1408 | 95.7 | Ophiocordyceps sinensis mitovirus 2 |
|  | k141_1252 | AZT88624.1 RNA-dependent RNA polymerase [Ophiocordyceps sinensis mitovirus 2] | 696 | 96.5 |  |
|  | k141_599 | QZE12022.1 MAG: RNA-dependent RNA polymerase [Sclerotinia sclerotiorum narnavirus 3] | 608 | 38.8 | Ophiocordyceps sinensis narnavirus 2 |
|  | k141_4202 | UNG44322.1 RNA-dependent RNA polymerase [Fusarium asiaticum narnavirus 1] | 485 | 53.1 | Ophiocordyceps sinensis narnavirus 2 |
|  | k141_3801 | QZE12024.1 MAG: RNA-dependent RNA polymerase [Sclerotinia sclerotiorum narnavirus 4] | 334 | 44.5 | Ophiocordyceps sinensis narnavirus 2 |
| SRR2533613 | k99_268 | AZT88623.1 RNA-dependent RNA polymerase [Ophiocordyceps sinensis mitovirus 1] | 2368 | 99 | Ophiocordyceps sinensis mitovirus 1 |
|  | k99_947 | AZT88624.1 RNA-dependent RNA polymerase [Ophiocordyceps sinensis mitovirus 2] | 2355 | 98.7 | Ophiocordyceps sinensis mitovirus 2 |
|  | k99_897 | XP_017607820.1 tobamovirus multiplication protein 2A [Gossypium arboreum] | 346 | 100 |  |
| SRR3658815 | k141_601 | QNQ74063.1 RdRp [Plasmopara viticola lesion associated orfanplasmovirus 1] | 2915 | 39 | Ophiocordyceps sinensis narnavirus 2 |
|  | k141_5051 | USW07202.1 putative RNA-dependent RNA polymerase [Plasmopara viticola lesion-associated ormycovirus 3] | 635 | 55.3 | Ophiocordyceps sinensis ormycovirus 1 |
| SRR3658816 | k141_414 | UUW20993.1 MAG: RNA-dependent RNA polymerase [Guiyang Paspalum thunbergii narna-like virus 1] | 3295 | 54 | Ophiocordyceps sinensis narnavirus 1 |
|  | k141_1655 | AZT88623.1 RNA-dependent RNA polymerase [Ophiocordyceps sinensis mitovirus 1] | 2549 | 50.7 | Ophiocordyceps sinensis mitovirus 3 |
|  | k141_1406 | AZT88624.1 RNA-dependent RNA polymerase [Ophiocordyceps sinensis mitovirus 2] | 2398 | 97.8 | Ophiocordyceps sinensis mitovirus 2 |
|  | k141_1584 | AZT88623.1 RNA-dependent RNA polymerase [Ophiocordyceps sinensis mitovirus 1] | 2299 | 98.6 | Ophiocordyceps sinensis mitovirus 1 |
|  | k141_1387 | QNQ74064.1 RdRp [Plasmopara viticola lesion associated orfanplasmovirus 2] | 1419 | 40 | Ophiocordyceps sinensis narnavirus 2 |
|  | k141_532 | QNQ74063.1 RdRp [Plasmopara viticola lesion associated orfanplasmovirus 1] | 1174 | 33.1 | Ophiocordyceps sinensis narnavirus 2 |
|  | k141_1412 | QED43022.1 RdRp, partial [Ophiocordyceps ourmiavirus A] | 1055 | 90.8 | Ophiocordyceps ourmiavirus A |
|  | k141_1562 | UYL95443.1 MAG: RNA-dependent RNA polymerase [Hulunbuir Botou tick virus 5] | 748 | 48.6 | Ophiocordyceps ourmiavirus A |
|  | k141_1094 | QIR30297.1 RNA-dependent RNA polymerase [Plasmopara viticola lesion associated narnavirus 18] | 511 | 39.3 |  |
| SRR3658817 | k141_7905 | USW07207.1 putative RNA-dependent RNA polymerase [Erysiphe lesion-associated ormycovirus 2] | 2420 | 44.7 | Ophiocordyceps sinensis ormycovirus 1 |
|  | k141_6550 | USW07212.1 hypothetical protein [Erysiphe lesion-associated ormycovirus 2] | 2110 | 34.6 | Ophiocordyceps sinensis ormycovirus 1 |
|  | k141_154 | UNG44322.1 RNA-dependent RNA polymerase [Fusarium asiaticum narnavirus 1] | 1489 | 41.1 | Ophiocordyceps sinensis narnavirus 2 |
|  | k141_182 | RKK53177.1 Retrovirus-related Pol polyprotein from transposon TNT 1-94 [Fusarium oxysporum] | 673 | 96.9 |  |
|  | k141_3062 | KAG7407040.1 Retrovirus-related Pol polyprotein from transposon TNT [Fusarium oxysporum f. sp. rapae] | 508 | 43 |  |
|  | k141_3925 | XP_018178050.1 herpesvirus latent membrane protein 1 (LMP1) domain-containing protein [Purpureocillium lilacinum] | 508 | 100 |  |
|  | k141_156 | KAG6996945.1 Retrovirus-related Pol polyprotein from transposon TNT [Fusarium oxysporum f. sp. conglutinans] | 329 | 90 |  |
| SRR5282569 | k141_569 | UYL95443.1 MAG: RNA-dependent RNA polymerase [Hulunbuir Botou tick virus 5] | 2900 | 51.7 | Ophiocordyceps ourmiavirus A |
|  | k141_3068 | QHD64819.1 RdRp [Erysiphe necator associated mitovirus 8] | 703 | 50.9 | Ophiocordyceps sinensis mitovirus 4 |
|  | k141_2396 | QHD64819.1 RdRp [Erysiphe necator associated mitovirus 8] | 603 | 63.2 | Ophiocordyceps sinensis mitovirus 4 |
|  | k141_1993 | UYL95349.1 MAG: RNA-dependent RNA polymerase, partial [Dali Mitov tick virus 1] | 306 | 75.2 |  |
| SRR5428527 | k119_1743 | QNQ74063.1 RdRp [Plasmopara viticola lesion associated orfanplasmovirus 1] | 2932 | 39 | Ophiocordyceps sinensis narnavirus 2 |
|  | k119_4140 | QHD64819.1 RdRp [Erysiphe necator associated mitovirus 8] | 2501 | 60.8 | Ophiocordyceps sinensis mitovirus 4 |
|  | k119_3852 | USW07207.1 putative RNA-dependent RNA polymerase [Erysiphe lesion-associated ormycovirus 2] | 2479 | 45.2 | Ophiocordyceps sinensis ormycovirus 1 |
|  | k119_5285 | BBU59838.1 RNA dependent RNA polymerase [Rosellinia necatrix partitivirus 15] | 2152 | 76.9 | Ophiocordyceps sinensis partitivirus 1 |
|  | k119_3637 | USW07212.1 hypothetical protein [Erysiphe lesion-associated ormycovirus 2] | 2143 | 34.4 |  |
|  | k119_6960 | UPT51545.1 RNA-dependent RNA polymerase [Oyster mushroom spherical virus] | 880 | 43.8 |  |
|  | k119_5440 | XP_039554243.1 BCL2/adenovirus E1B 19 kDa protein-interacting protein 3-like isoform X2 [Passer montanus] | 736 | 100 |  |
|  | k119_1333 | XP_052611577.1 endogenous retrovirus group K member 24 Gag polyprotein-like [Peromyscus californicus insignis] | 539 | 57.6 |  |
|  | k119_4390 | BBD71147.1 putative RNA-dependent RNA polymerase [Alternaria alternata fusarivirus 1] | 515 | 36.6 |  |
|  | k119_3337 | BDF97667.1 RNA-dependent RNA polymerase [Penicillium vanoranjei associated RNA virus 1] | 477 | 75.8 |  |
|  | k119_5505 | UJT31894.1 RNA-dependent RNA polymerase, partial [Picobirnavirus sp.] | 454 | 100 |  |
|  | k119_6988 | XP_008821367.1 BCL2/adenovirus E1B 19 kDa protein-interacting protein 3 [Nannospalax galili] | 426 | 98.6 |  |
|  | k119_4626 | YP_009143301.1 polyprotein [Sclerotinia sclerotiorum fusarivirus 1] | 371 | 35.8 |  |
|  | k119_564 | BDF97667.1 RNA-dependent RNA polymerase [Penicillium vanoranjei associated RNA virus 1] | 341 | 69.9 |  |
|  | k119_4003 | NP_783203.1 RNA-dependent RNA polymerase [Oyster mushroom spherical virus] | 338 | 60.6 |  |
| SRR5446809 | k141_4520 | USW07207.1 putative RNA-dependent RNA polymerase [Erysiphe lesion-associated ormycovirus 2] | 2439 | 45.2 | Ophiocordyceps sinensis ormycovirus 1 |
|  | k141_3105 | USW07212.1 hypothetical protein [Erysiphe lesion-associated ormycovirus 2] | 2130 | 34.7 |  |
|  | k141_4441 | YP_010798347.1 MAG: hypothetical protein QKN93_gp2 [Narnavirus sp.] | 1437 | 38.9 |  |
|  | k141_3745 | QED43117.1 CP [Garlic common latent virus] | 809 | 100 |  |
|  | k141_2446 | QED43091.1 TGB2 [Garlic common latent virus] | 490 | 100 |  |
|  | k141_2393 | QED43161.1 replicase [Garlic common latent virus] | 477 | 100 |  |
|  | k141_2118 | QED43119.1 replicase [Garlic common latent virus] | 314 | 100 |  |
| SRR8258357 | k141_7072 | QKN22686.1 RNA-dependent RNA polymerase [Erysiphe necator associated flexivirus 1] | 7877 | 56.9 | Ophiocordyceps sinensis deltaflexivirus 1 |
|  | k141_3254 | BED98313.1 MAG: hypothetical protein [Aspergillus flavus vivivirus 1] | 2482 | 26.9 | Ophiocordyceps sinensis vivivirus 1 |
|  | k141_30 | USW07207.1 putative RNA-dependent RNA polymerase [Erysiphe lesion-associated ormycovirus 2] | 2353 | 45.2 | Ophiocordyceps sinensis ormycovirus 1 |
|  | k141_10787 | USW07212.1 hypothetical protein [Erysiphe lesion-associated ormycovirus 2] | 2095 | 34.1 |  |
|  | k141_2485 | QYJ09848.1 MAG: RNA-dependent RNA polymerase, partial [Sisal-associated virgavirus A] | 1276 | 51.9 | Ophiocordyceps sinensis vivivirus 1 |
|  | k141_7781 | QED43022.1 RdRp, partial [Ophiocordyceps ourmiavirus A] | 964 | 90.5 | Ophiocordyceps ourmiavirus A |
|  | k141_4704 | QHD64819.1 RdRp [Erysiphe necator associated mitovirus 8] | 909 | 66.1 | Ophiocordyceps sinensis mitovirus 4 |
|  | k141_960 | UVG42310.1 hypothetical protein USA: Philadelphia, PA_000011 [unidentified adenovirus] | 707 | 98.2 |  |
|  | k141_6056 | QYD13422.1 MAG: putative RNA-dependent RNA polymerase, partial [Sisal-associated Virgavirus C] | 618 | 39.6 |  |
|  | k141_4561 | QED43022.1 RdRp, partial [Ophiocordyceps ourmiavirus A] | 616 | 97.2 |  |
|  | k141_11115 | QYD13422.1 MAG: putative RNA-dependent RNA polymerase, partial [Sisal-associated Virgavirus C] | 491 | 60.3 |  |
|  | k141_3006 | QYD13422.1 MAG: putative RNA-dependent RNA polymerase, partial [Sisal-associated Virgavirus C] | 469 | 47.2 |  |
|  | k141_1723 | UUW20993.1 MAG: RNA-dependent RNA polymerase [Guiyang Paspalum thunbergii narna-like virus 1] | 459 | 55 | Ophiocordyceps sinensis narnavirus 1 |
|  | k141_157 | QJT93762.1 RNA-dependent RNA polymerase [Erysiphe necator associated narnavirus 30] | 451 | 44.2 | Ophiocordyceps sinensis narnavirus 4 |
|  | k141_4629 | YP_009333139.1 RNA-dependent RNA polymerase [Beihai narna-like virus 22] | 418 | 35.1 |  |
|  | k141_3610 | QKN22726.1 replicase [Erysiphe necator associated ssRNA virus 12] | 373 | 64.3 |  |
|  | k141_9930 | QHD64819.1 RdRp [Erysiphe necator associated mitovirus 8] | 327 | 39.5 |  |
|  | k141_4230 | QYJ09848.1 MAG: RNA-dependent RNA polymerase, partial [Sisal-associated virgavirus A] | 307 | 75.5 |  |
|  | k141_4364 | QYD13422.1 MAG: putative RNA-dependent RNA polymerase, partial [Sisal-associated Virgavirus C] | 303 | 53.1 |  |
| SRR9290661 | k141_6035 | QIR30286.1 RNA-dependent RNA polymerase [Plasmopara viticola lesion associated narnavirus 7] | 2001 | 41 | Ophiocordyceps sinensis narnavirus 4 |
|  | k141_5880 | BCX55510.1 putative RNA-dependent RNA polymerase [Cryphonectria naterciae splipalmivirus 1] | 1124 | 33.7 | Ophiocordyceps sinensis narnavirus 3 |
|  | k141_2305 | UYL94525.1 MAG: RNA-dependent RNA polymerase [Leptosphaeria biglobosa narnavirus 7] | 956 | 32.7 | Ophiocordyceps sinensis narnavirus 3 |
|  | k141_9341 | QED43022.1 RdRp, partial [Ophiocordyceps ourmiavirus A] | 549 | 80.2 | Ophiocordyceps ourmiavirus A |
|  | k141_1335 | QHD64819.1 RdRp [Erysiphe necator associated mitovirus 8] | 534 | 61 | Ophiocordyceps sinensis mitovirus 4 |
|  | k141_8284 | QHD64819.1 RdRp [Erysiphe necator associated mitovirus 8] | 434 | 67.1 |  |
|  | k141_8513 | AHF48631.1 RNA-dependent RNA polymerase [Sclerotinia sclerotiorum mitovirus 15] | 395 | 49.6 | Ophiocordyceps sinensis mitovirus 3 |
