# Supplementary material for: Unveiling mycoviral diversity in Ophiocordyceps sinensis through transcriptome analyses
Source: Front Microbiol. 2024 Nov 25;15:1493365. doi: 10.3389/fmicb.2024.1493365 (PMC11625762; doi:10.3389/fmicb.2024.1493365)
Supplement: Supplementary Table S4 — Coverage (% genome length) of each mycovirus identified in the public transcriptome of different O. sinensis strains. [file Table_4.docx]

Table S4 Coverage (% genome length) of each mycovirus identified in the public transcriptome of different *O. sinensis* strains.

| coverage (% genome  length)  SRR ID | OsOMV1 | OsOVA | OsMV1 | OsMV2 | OsMV3 | OsMV4 | OsNV1 | OsNV2 | OsNV3 | OsNV4 | OsPV1 | OsVV1 | OsDFV1 |
| --- | --- | --- | --- | --- | --- | --- | --- | --- | --- | --- | --- | --- | --- |
| SRR11548640 |  |  |  | 98.2 | 98.7 |  |  |  |  |  |  |  |  |
| SRR12952889 | 30.3 | 21.6 |  |  | 67.1 |  | 27.2 |  |  |  |  |  | 99.7 |
| SRR13286702 | 19.2 |  |  |  |  |  |  |  |  |  |  |  |  |
| SRR21290686 | 96.6 |  |  | 57.7 | 96.2 |  |  | 20.4 |  |  |  |  |  |
| SRR2533613 |  |  | 99.2 | 96.6 |  |  |  |  |  |  |  |  |  |
| SRR3658815 | 25.3 |  |  |  |  |  |  | 98.0 |  |  |  |  |  |
| SRR3658816 |  | 35.6 | 96.4 | 98.3 | 98.0 |  | 97.2 | 47.7 |  |  |  |  |  |
| SRR3658817 | 96.6 |  |  |  |  |  |  | 50.1 |  |  |  |  |  |
| SRR5282569 |  | 97.7 |  |  |  | 28.1 |  |  |  |  |  |  |  |
| SRR5428527 | 98.9 |  |  |  |  | 100 |  | 98.6 |  |  | 100 |  |  |
| SRR5446809 | 97.3 |  |  |  |  |  |  |  |  |  |  |  |  |
| SRR8258357 | 93.9 | 32.5 |  |  |  | 36.3 | 13.5 |  |  | 22.2 |  | 100 | 100 |
| SRR9290661 |  | 18.5 |  |  | 15.2 | 21.4 |  |  | 51.7 | 98.6 |  |  |  |

OsOMV1, Ophiocordyceps sinensis ormycovirus 1; OsOVA, Ophiocordyceps ourmiavirus A; OsMV, Ophiocordyceps sinensis mitovirus; OsNV, Ophiocordyceps sinensis narnavirus; OsPV1, Ophiocordyceps sinensis partitivirus 1; OsVV1, Ophiocordyceps sinensis vivivirus 1; OsDFV1, Ophiocordyceps sinensis deltaflexivirus 1.
