# Supplementary material for: Unveiling mycoviral diversity in Ophiocordyceps sinensis through transcriptome analyses
Source: Front Microbiol. 2024 Nov 25;15:1493365. doi: 10.3389/fmicb.2024.1493365 (PMC11625762; doi:10.3389/fmicb.2024.1493365)
Supplement: Supplementary Table S5 — Coverage (% genome length) of each mycovirus identified in the public transcriptome of Chinese cordyceps at different developmental stages. [file Table_5.docx]

Table S5 Coverage (% genome length) of each mycovirus identified in the public transcriptome of Chinese cordyceps at different developmental stages.

|  | SRA ID | Tissue | OsOMV1 | OsOVA | OsMV1 | OsMV2 | OsMV3 | OsMV4 | OsNV1 | OsNV2 | OsNV3 | OsNV4 | OsPV1 | OsVV1 | OsDFV1 |
| --- | --- | --- | --- | --- | --- | --- | --- | --- | --- | --- | --- | --- | --- | --- | --- |
| PRJNA  507459  (Li et al., 2019) | SRR8258340 | hyphae | 30.6 | 40.1 | 33.1 | 26.3 | 97.6 |  |  | 29.5 | 96.6 | 96.1 |  |  |  |
|  | SRR8258343 | sclerotium | 97.7 | 32.9 |  |  | 70.7 |  | 73.0 |  |  |  |  |  |  |
|  | SRR8258346 | primordium |  | 95.4 |  |  |  |  | 47.4 |  |  | 96.5 |  |  | 97.7 |
|  | SRR8258349 | young fruitbody | 31.6 | 54.8 |  |  |  |  | 73.5 | 13.7 | 96.9 | 90.2 |  |  | 6.8 |
|  | SRR8258352 | developed fruitbody | 97.0 | 97.2 |  |  | 86.8 |  | 94.0 |  | 31.9 | 96.6 |  |  | 70.3 |
|  | SRR8258357 | mature fruitbody | 93.9 | 32.5 |  |  |  | 36.3 | 13.5 |  |  | 22.2 |  | 100 | 100 |
| PRJNA  673413  Stain  SICC 5.02  (Zhang et al., 2021) | SRR12952889 | mycoparasite complex | 30.3 | 21.6 |  |  | 67.1 |  | 27.2 |  |  |  |  |  | 99.7 |
|  | SRR12952892 | sclerotium | 51.3 |  |  |  |  |  |  | 19.4 |  |  |  |  |  |
|  | SRR12952895 | fruitbody |  |  |  |  |  |  |  |  |  |  |  |  |  |
| PRJNA  625214  Stain  IOZ07  (Li et al., 2020) | SRR11548640 | cultured mycelia |  |  |  | 98.2 | 98.7 |  |  |  |  |  |  |  |  |
|  | SRR11547913 | Blastospores in proliferative stage | 24.7 |  |  | 71.0 |  |  | 99.8 |  |  |  |  |  |  |
|  | SRR11547906 | Blastospores in stationary stage | 44.0 |  |  | 51.5 | 19.6 |  | 99.8 | 97.2 |  |  |  |  |  |
|  | SRR11547905 | Prehyphae | 20.3 |  |  |  |  |  |  |  |  |  |  |  |  |
|  | SRR11547910 | Hyphae | 18.7 |  |  |  |  |  | 94.4 |  |  |  |  |  |  |
| PRJNA  600609  Stain  IOZ07  (Zhao et al., 2020) | SRR10878126 | sclerotium | 98.6 | 97.3 |  |  |  |  |  | 37.1 |  |  |  |  |  |
|  | SRR10878121 | primordium | 93.5 |  |  |  |  |  |  | 34.1 |  |  |  |  |  |

OsOMV1, Ophiocordyceps sinensis ormycovirus 1; OsOVA, Ophiocordyceps ourmiavirus A; OsMV, Ophiocordyceps sinensis mitovirus; OsNV, Ophiocordyceps sinensis narnavirus; OsPV1, Ophiocordyceps sinensis partitivirus 1; OsVV1, Ophiocordyceps sinensis vivivirus 1; OsDFV1, Ophiocordyceps sinensis deltaflexivirus 1.
