# Supplementary material for: Unveiling mycoviral diversity in Ophiocordyceps sinensis through transcriptome analyses
Source: Front Microbiol. 2024 Nov 25;15:1493365. doi: 10.3389/fmicb.2024.1493365 (PMC11625762; doi:10.3389/fmicb.2024.1493365)
Supplement: Supplementary Table S6 — Detailed information on contigs obtained from 6 different samples by analyzing the PRJNA507459. [file Table_6.docx]

Table S6 Detailed information on contigs obtained from 6 different samples by analyzing the PRJNA507459.

| SRA ID | contig | protein description | Length (nt) | Ident (%) | Name of putative virus |
| --- | --- | --- | --- | --- | --- |
| SRR8258340 | k141_6866 | AZT88623.1 RNA-dependent RNA polymerase [Ophiocordyceps sinensis mitovirus 1] | 2537 | 42.4 | Ophiocordyceps sinensis mitovirus 3 |
|  | k141_7821 | UYL94524.1 MAG: hypothetical protein [Leptosphaeria biglobosa narnavirus 6] | 2100 | 52 | Ophiocordyceps sinensis narnavirus 3 |
|  | k141_7822 | QIR30286.1 RNA-dependent RNA polymerase [Plasmopara viticola lesion associated narnavirus 7] | 1950 | 40 | Ophiocordyceps sinensis narnavirus 4 |
|  | k141_5592 | USW07212.1 hypothetical protein [Erysiphe lesion-associated ormycovirus 2] | 2068 | 34.3 | Ophiocordyceps sinensis ormycovirus 1 |
|  | k141_8187 | UYL95443.1 MAG: RNA-dependent RNA polymerase [Hulunbuir Botou tick virus 5] | 1190 | 50.4 | Ophiocordyceps ourmiavirus A |
|  | k141_753 | QZE12022.1 MAG: RNA-dependent RNA polymerase [Sclerotinia sclerotiorum narnavirus 3] | 878 | 40.9 | Ophiocordyceps sinensis narnavirus 2 |
|  | k141_8135 | AZT88623.1 RNA-dependent RNA polymerase [Ophiocordyceps sinensis mitovirus 1] | 790 | 98.2 | Ophiocordyceps sinensis mitovirus 1 |
|  | k141_7417 | USW07207.1 putative RNA-dependent RNA polymerase [Erysiphe lesion-associated ormycovirus 2] | 767 | 39.3 | Ophiocordyceps sinensis ormycovirus 1 |
|  | k141_3686 | UYL95443.1 MAG: RNA-dependent RNA polymerase [Hulunbuir Botou tick virus 5] | 701 | 51.3 |  |
|  | k141_7143 | USW07202.1 putative RNA-dependent RNA polymerase [Plasmopara viticola lesion-associated ormycovirus 3] | 679 | 54.4 |  |
|  | k141_964 | USW07207.1 putative RNA-dependent RNA polymerase [Erysiphe lesion-associated ormycovirus 2] | 660 | 42.5 |  |
|  | k141_976 | AZT88624.1 RNA-dependent RNA polymerase [Ophiocordyceps sinensis mitovirus 2] | 641 | 95.8 | Ophiocordyceps sinensis mitovirus 2 |
|  | k141_7705 | QNQ74066.1 RdRp [Plasmopara viticola lesion associated orfanplasmovirus 4] | 603 | 39.3 | Ophiocordyceps sinensis narnavirus 2 |
|  | k141_3521 | QNQ74065.1 RdRp [Plasmopara viticola lesion associated orfanplasmovirus 3] | 396 | 33.9 | Ophiocordyceps sinensis narnavirus 2 |
|  | k141_8354 | AZT88623.1 RNA-dependent RNA polymerase [Ophiocordyceps sinensis mitovirus 1] | 302 | 95.5 |  |
| SRR8258343 | k141_14300 | UUW20993.1 MAG: RNA-dependent RNA polymerase [Guiyang Paspalum thunbergii narna-like virus 1] | 2476 | 54.1 | Ophiocordyceps sinensis narnavirus 1 |
|  | k141_13907 | USW07207.1 putative RNA-dependent RNA polymerase [Erysiphe lesion-associated ormycovirus 2] | 2448 | 44.6 | Ophiocordyceps sinensis ormycovirus 1 |
|  | k141_2810 | USW07212.1 hypothetical protein [Erysiphe lesion-associated ormycovirus 2] | 2092 | 33.6 |  |
|  | k141_179 | AHF48631.1 RNA-dependent RNA polymerase [Sclerotinia sclerotiorum mitovirus 15] | 1838 | 52.8 | Ophiocordyceps sinensis mitovirus 3 |
|  | k141_62 | QED43022.1 RdRp, partial [Ophiocordyceps ourmiavirus A] | 977 | 89.9 | Ophiocordyceps ourmiavirus A |
|  | k141_4198 | PNF33053.1 Retrovirus-related Pol polyprotein from transposon TNT 1-94 [Cryptotermes secundus] | 636 | 46.8 |  |
|  | k141_2744 | AZT88623.1 RNA-dependent RNA polymerase [Ophiocordyceps sinensis mitovirus 1] | 598 | 42.7 | Ophiocordyceps sinensis mitovirus 3 |
|  | k141_3236 | UYL95443.1 MAG: RNA-dependent RNA polymerase [Hulunbuir Botou tick virus 5] | 542 | 65.5 |  |
| SRR8258346 | k141_7831 | QKN22686.1 RNA-dependent RNA polymerase [Erysiphe necator associated flexivirus 1] | 7696 | 56.9 | Ophiocordyceps sinensis deltaflexivirus 1 |
|  | k141_5503 | UYL95443.1 MAG: RNA-dependent RNA polymerase [Hulunbuir Botou tick virus 5] | 2832 | 52.3 | Ophiocordyceps ourmiavirus A |
|  | k141_12307 | QIR30286.1 RNA-dependent RNA polymerase [Plasmopara viticola lesion associated narnavirus 7] | 1957 | 41 | Ophiocordyceps sinensis narnavirus 4 |
|  | k141_11859 | UUW20993.1 MAG: RNA-dependent RNA polymerase [Guiyang Paspalum thunbergii narna-like virus 1] | 1606 | 54.6 | Ophiocordyceps sinensis narnavirus 1 |
|  | k141_11309 | YP_010804003.1 ribose-phosphate pyrophosphokinase [Fadolivirus 1] | 565 | 52.4 |  |
|  | k141_11164 | UUW20993.1 MAG: RNA-dependent RNA polymerase [Guiyang Paspalum thunbergii narna-like virus 1] | 450 | 61.1 |  |
|  | k141_7035 | YP_010804235.1 hypothetical protein QKU48_gp1352 [Fadolivirus 1] | 408 | 46.8 |  |
|  | k141_317 | YP_010804003.1 ribose-phosphate pyrophosphokinase [Fadolivirus 1] | 385 | 68.9 |  |
|  | k141_3667 | UGN74103.1 structural protein, partial [Adenovirus sp.] | 346 | 100 |  |
|  | k141_7451 | AYV82990.1 MAG: chromosome condensation regulator [Hyperionvirus sp.] | 324 | 35 |  |
| SRR8258349 | k141_7763 | UUW20993.1 MAG: RNA-dependent RNA polymerase [Guiyang Paspalum thunbergii narna-like virus 1] | 2493 | 54.5 | Ophiocordyceps sinensis narnavirus 1 |
|  | k141_4175 | UYL94525.1 MAG: RNA-dependent RNA polymerase [Leptosphaeria biglobosa narnavirus 7] | 2106 | 32.8 | Ophiocordyceps sinensis narnavirus 3 |
|  | k141_925 | QIR30286.1 RNA-dependent RNA polymerase [Plasmopara viticola lesion associated narnavirus 7] | 1831 | 39.3 | Ophiocordyceps sinensis narnavirus 4 |
|  | k141_695 | QED43022.1 RdRp, partial [Ophiocordyceps ourmiavirus A] | 1627 | 92 | Ophiocordyceps ourmiavirus A |
|  | k141_6250 | USW07207.1 putative RNA-dependent RNA polymerase [Erysiphe lesion-associated ormycovirus 2] | 792 | 45 | Ophiocordyceps sinensis ormycovirus 1 |
|  | k141_1754 | UYL95443.1 MAG: RNA-dependent RNA polymerase [Hulunbuir Botou tick virus 5] | 661 | 50.9 |  |
|  | k141_6337 | USW07202.1 putative RNA-dependent RNA polymerase [Plasmopara viticola lesion-associated ormycovirus 3] | 589 | 53.5 |  |
|  | k141_3637 | QKN22648.1 hypothetical protein [Erysiphe necator associated deltaflexivirus 2] | 533 | 58 | Ophiocordyceps sinensis deltaflexivirus 1 |
|  | k141_7613 | USW07203.1 hypothetical protein [Plasmopara viticola lesion-associated ormycovirus 3] | 519 | 42.4 |  |
|  | k141_2680 | QKN22686.1 RNA-dependent RNA polymerase [Erysiphe necator associated flexivirus 1] | 506 | 54.2 |  |
|  | k141_1081 | QKN22686.1 RNA-dependent RNA polymerase [Erysiphe necator associated flexivirus 1] | 478 | 70.4 |  |
|  | k141_4305 | UMO78240.1 hypothetical protein [Pandoravirus belohorizontensis] | 461 | 43.4 |  |
|  | k141_7037 | UJT31894.1 RNA-dependent RNA polymerase, partial [Picobirnavirus sp.] | 460 | 97.9 |  |
|  | k141_2093 | QBZ81702.1 hypothetical protein pclt_cds_1119 [Pandoravirus celtis] | 439 | 33.6 |  |
|  | k141_4257 | UNG44322.1 RNA-dependent RNA polymerase [Fusarium asiaticum narnavirus 1] | 409 | 58.8 | Ophiocordyceps sinensis narnavirus 2 |
|  | k141_2038 | BED98292.1 MAG: RNA-dependent RNA polymerase [Aspergillus flavus deltaflexivirus 1] | 345 | 71.1 |  |
|  | k141_7331 | CAH6421454.1 Phosphoribosylpyrophosphate synthetase [uncultured virus] | 344 | 53.1 |  |
|  | k141_348 | QTH80200.1 MAG: RNA-dependent RNA polymerase [Pestalotiopsis deltaflexivirus 1] | 341 | 64 |  |
|  | k141_3102 | QZE12024.1 MAG: RNA-dependent RNA polymerase [Sclerotinia sclerotiorum narnavirus 4] | 326 | 54.9 | Ophiocordyceps sinensis narnavirus 2 |
| SRR8258352 | k141_6744 | QKN22686.1 RNA-dependent RNA polymerase [Erysiphe necator associated flexivirus 1] | 5541 | 34.2 | Ophiocordyceps sinensis deltaflexivirus 1 |
|  | k141_8765 | UUW20993.1 MAG: RNA-dependent RNA polymerase [Guiyang Paspalum thunbergii narna-like virus 1] | 3189 | 43 | Ophiocordyceps sinensis narnavirus 1 |
|  | k141_5261 | UYL95443.1 MAG: RNA-dependent RNA polymerase [Hulunbuir Botou tick virus 5] | 2884 | 37.4 | Ophiocordyceps ourmiavirus A |
|  | k141_9446 | USW07207.1 putative RNA-dependent RNA polymerase [Erysiphe lesion-associated ormycovirus 2] | 2430 | 34.5 |  |
|  | k141_5536 | AZT88623.1 RNA-dependent RNA polymerase [Ophiocordyceps sinensis mitovirus 1] | 2256 | 60.6 | Ophiocordyceps sinensis mitovirus 3 |
|  | k141_1442 | USW07212.1 hypothetical protein [Erysiphe lesion-associated ormycovirus 2] | 2096 | 45.7 | Ophiocordyceps sinensis ormycovirus 1 |
|  | k141_10702 | QIR30286.1 RNA-dependent RNA polymerase [Plasmopara viticola lesion associated narnavirus 7] | 1961 | 53.9 | Ophiocordyceps sinensis narnavirus 4 |
|  | k141_7619 | BDQ13824.1 unnamed protein product [Fusarium deltaflexivirus 2] | 1640 | 58.1 |  |
|  | k141_3695 | AHF48631.1 RNA-dependent RNA polymerase [Sclerotinia sclerotiorum mitovirus 15] | 1005 | 40.9 | Ophiocordyceps sinensis mitovirus 3 |
|  | k141_1981 | UYL94524.1 MAG: hypothetical protein [Leptosphaeria biglobosa narnavirus 6] | 694 | 52 | Ophiocordyceps sinensis narnavirus 3 |
|  | k141_5196 | QKV51041.1 putative capsid protein [Crucivirus sp.] | 392 | 53.5 |  |
|  | k141_10110 | UYL94525.1 MAG: RNA-dependent RNA polymerase [Leptosphaeria biglobosa narnavirus 7] | 330 | 51.6 | Ophiocordyceps sinensis narnavirus 3 |
| SRR8258357 | k141_7072 | QKN22686.1 RNA-dependent RNA polymerase [Erysiphe necator associated flexivirus 1] | 7877 | 56.9 | Ophiocordyceps sinensis deltaflexivirus 1 |
|  | k141_3254 | BED98313.1 MAG: hypothetical protein [Aspergillus flavus vivivirus 1] | 2482 | 26.9 | Ophiocordyceps sinensis vivivirus 1 |
|  | k141_30 | USW07207.1 putative RNA-dependent RNA polymerase [Erysiphe lesion-associated ormycovirus 2] | 2353 | 45.2 | Ophiocordyceps sinensis ormycovirus 1 |
|  | k141_10787 | USW07212.1 hypothetical protein [Erysiphe lesion-associated ormycovirus 2] | 2095 | 34.1 |  |
|  | k141_2485 | QYJ09848.1 MAG: RNA-dependent RNA polymerase, partial [Sisal-associated virgavirus A] | 1276 | 51.9 | Ophiocordyceps sinensis vivivirus 1 |
|  | k141_7781 | QED43022.1 RdRp, partial [Ophiocordyceps ourmiavirus A] | 964 | 90.5 | Ophiocordyceps ourmiavirus A |
|  | k141_4704 | QHD64819.1 RdRp [Erysiphe necator associated mitovirus 8] | 909 | 66.1 | Ophiocordyceps sinensis mitovirus 4 |
|  | k141_960 | UVG42310.1 hypothetical protein USA: Philadelphia, PA_000011 [unidentified adenovirus] | 707 | 98.2 |  |
|  | k141_6056 | QYD13422.1 MAG: putative RNA-dependent RNA polymerase, partial [Sisal-associated Virgavirus C] | 618 | 39.6 |  |
|  | k141_4561 | QED43022.1 RdRp, partial [Ophiocordyceps ourmiavirus A] | 616 | 97.2 |  |
|  | k141_11115 | QYD13422.1 MAG: putative RNA-dependent RNA polymerase, partial [Sisal-associated Virgavirus C] | 491 | 60.3 |  |
|  | k141_3006 | QYD13422.1 MAG: putative RNA-dependent RNA polymerase, partial [Sisal-associated Virgavirus C] | 469 | 47.2 |  |
|  | k141_1723 | UUW20993.1 MAG: RNA-dependent RNA polymerase [Guiyang Paspalum thunbergii narna-like virus 1] | 459 | 55 | Ophiocordyceps sinensis narnavirus 1 |
|  | k141_157 | QJT93762.1 RNA-dependent RNA polymerase [Erysiphe necator associated narnavirus 30] | 451 | 44.2 | Ophiocordyceps sinensis narnavirus 4 |
|  | k141_4629 | YP_009333139.1 RNA-dependent RNA polymerase [Beihai narna-like virus 22] | 418 | 35.1 |  |
|  | k141_3610 | QKN22726.1 replicase [Erysiphe necator associated ssRNA virus 12] | 373 | 64.3 |  |
|  | k141_9930 | QHD64819.1 RdRp [Erysiphe necator associated mitovirus 8] | 327 | 39.5 |  |
|  | k141_4230 | QYJ09848.1 MAG: RNA-dependent RNA polymerase, partial [Sisal-associated virgavirus A] | 307 | 75.5 |  |
|  | k141_4364 | QYD13422.1 MAG: putative RNA-dependent RNA polymerase, partial [Sisal-associated Virgavirus C] | 303 | 53.1 |  |
