# Supplementary material for: Unveiling mycoviral diversity in Ophiocordyceps sinensis through transcriptome analyses
Source: Front Microbiol. 2024 Nov 25;15:1493365. doi: 10.3389/fmicb.2024.1493365 (PMC11625762; doi:10.3389/fmicb.2024.1493365)
Supplement: Supplementary Table S7 — Detailed information on contigs obtained from 3 different samples by analyzing the PRJNA673413. [file Table_7.docx]

Table S7 Detailed information on contigs obtained from 3 different samples by analyzing the PRJNA673413.

| SRA ID | contig | protein description | Length (nt) | Ident (%) | Name of putative virus |
| --- | --- | --- | --- | --- | --- |
| SRR12952889 | k141_1472 | QKN22686.1 RNA-dependent RNA polymerase [Erysiphe necator associated flexivirus 1] | 7850 | 56.9 | Ophiocordyceps sinensis deltaflexivirus 1 |
|  | k141_1605 | AHF48631.1 RNA-dependent RNA polymerase [Sclerotinia sclerotiorum mitovirus 15] | 1744 | 53.6 | Ophiocordyceps sinensis mitovirus 3 |
|  | k141_2172 | UUW20993.1 MAG: RNA-dependent RNA polymerase [Guiyang Paspalum thunbergii narna-like virus 1] | 923 | 48.1 | Ophiocordyceps sinensis narnavirus 1 |
|  | k141_3419 | UUW20993.1 MAG: RNA-dependent RNA polymerase [Guiyang Paspalum thunbergii narna-like virus 1] | 898 | 65.8 |  |
|  | k141_2958 | UUW20993.1 MAG: RNA-dependent RNA polymerase [Guiyang Paspalum thunbergii narna-like virus 1] | 856 | 52.1 |  |
|  | k141_1653 | USW07202.1 putative RNA-dependent RNA polymerase [Plasmopara viticola lesion-associated ormycovirus 3] | 760 | 46.6 | Ophiocordyceps sinensis ormycovirus 1 |
|  | k141_2125 | USW07204.1 putative RNA-dependent RNA polymerase [Erysiphe lesion-associated ormycovirus 3] | 733 | 44.8 |  |
|  | k141_276 | UYL95443.1 MAG: RNA-dependent RNA polymerase [Hulunbuir Botou tick virus 5] | 642 | 51.9 | Ophiocordyceps ourmiavirus A |
|  | k141_1296 | AZT88623.1 RNA-dependent RNA polymerase [Ophiocordyceps sinensis mitovirus 1] | 608 | 46.1 | Ophiocordyceps sinensis mitovirus 3 |
|  | k141_1007 | USW07207.1 putative RNA-dependent RNA polymerase [Erysiphe lesion-associated ormycovirus 2] | 530 | 50.9 |  |
|  | k141_3613 | UPO93688.1 coat protein [Metarhizium brunneum bipartite mycovirus 1] | 521 | 76.3 |  |
|  | k141_1703 | USW07207.1 putative RNA-dependent RNA polymerase [Erysiphe lesion-associated ormycovirus 2] | 478 | 41.6 |  |
|  | k141_3924 | USW07212.1 hypothetical protein [Erysiphe lesion-associated ormycovirus 2] | 398 | 42.1 |  |
|  | k141_2872 | ELU41375.1 pneumovirus matrix domain-containing protein [Rhizoctonia solani AG-1 IA] | 348 | 77.8 |  |
|  | k141_495 | USW07203.1 hypothetical protein [Plasmopara viticola lesion-associated ormycovirus 3] | 328 | 42.1 |  |
| SRR12952892 | k141_831 | USW07202.1 putative RNA-dependent RNA polymerase [Plasmopara viticola lesion-associated ormycovirus 3] | 1286 | 50.7 | Ophiocordyceps sinensis ormycovirus 1 |
|  | k141_4215 | USW07207.1 putative RNA-dependent RNA polymerase [Erysiphe lesion-associated ormycovirus 2] | 626 | 47.9 |  |
|  | k141_1411 | QZE12024.1 MAG: RNA-dependent RNA polymerase [Sclerotinia sclerotiorum narnavirus 4] | 578 | 47.7 | Ophiocordyceps sinensis narnavirus 2 |
|  | k141_4013 | XP_024500291.1 Influenza virus NS1A-binding protein [Strongyloides ratti] | 536 | 62.8 |  |
|  | k141_2587 | USW07203.1 hypothetical protein [Plasmopara viticola lesion-associated ormycovirus 3] | 471 | 40.8 |  |
|  | k141_6082 | XP_024500291.1 Influenza virus NS1A-binding protein [Strongyloides ratti] | 409 | 69.6 |  |
|  | k141_5974 | XP_024506800.1 Xenotropic and polytropic retrovirus receptor 1 [Strongyloides ratti] | 366 | 73 |  |
|  | k141_5311 | USW07212.1 hypothetical protein [Erysiphe lesion-associated ormycovirus 2] | 348 | 38.6 |  |
| SRR12952895 | k141_540 | QLC27601.1 putative methyltransferase [Erysiphe necator associated abispo virus 2] | 648 | 40.9 |  |
|  | k141_15 | UJT31894.1 RNA-dependent RNA polymerase, partial [Picobirnavirus sp.] | 381 | 100 |  |
