# Supplementary material for: Unveiling mycoviral diversity in Ophiocordyceps sinensis through transcriptome analyses
Source: Front Microbiol. 2024 Nov 25;15:1493365. doi: 10.3389/fmicb.2024.1493365 (PMC11625762; doi:10.3389/fmicb.2024.1493365)
Supplement: Supplementary Table S8 — Detailed information on contigs obtained from 5 different samples by analyzing the PRJNA625214. [file Table_8.docx]

Table S8 Detailed information on contigs obtained from 5 different samples by analyzing the PRJNA625214.

| SRA ID | contig | protein description | Length (nt) | Ident (%) | Name of putative virus |
| --- | --- | --- | --- | --- | --- |
| SRR11548640 | k141_726 | AZT88623.1 RNA-dependent RNA polymerase [Ophiocordyceps sinensis mitovirus 1] | 2565 | 50.5 | Ophiocordyceps sinensis mitovirus 3 |
|  | k141_187 | AZT88624.1 RNA-dependent RNA polymerase [Ophiocordyceps sinensis mitovirus 2] | 2396 | 97.9 | Ophiocordyceps sinensis mitovirus 2 |
| SRR11547913 | k141_2600 | UUW20993.1 MAG: RNA-dependent RNA polymerase [Guiyang Paspalum thunbergii narna-like virus 1 | 3386 | 53.8 | Ophiocordyceps sinensis narnavirus 1 |
|  | k141_1473 | AZT88624.1 RNA-dependent RNA polymerase [Ophiocordyceps sinensis mitovirus 2] | 1732 | 96 | Ophiocordyceps sinensis mitovirus 2 |
|  | k141_7121 | YP_010798565.1 glycoprotein [Lepidopteran rhabdo-related virus OKIAV34] | 1498 | 42.4 |  |
|  | k141_300 | YP_010798562.1 nucleocapsid protein [Lepidopteran rhabdo-related virus OKIAV34] | 969 | 44.3 |  |
|  | k141_365 | GBP52898.1 Retrovirus-related Pol polyprotein from type-1 retrotransposable element R1 [Eumeta japonica] | 676 | 47.8 |  |
|  | k141_3245 | USW07205.1 hypothetical protein [Erysiphe lesion-associated ormycovirus 3] | 533 | 37.1 | Ophiocordyceps sinensis ormycovirus 1 |
|  | k141_2840 | AZT88624.1 RNA-dependent RNA polymerase [Ophiocordyceps sinensis mitovirus 2] | 472 | 98.1 |  |
|  | k141_5977 | GBP63085.1 Retrovirus-related Pol polyprotein from transposon TNT 1-94 [Eumeta japonica] | 398 | 68.4 |  |
|  | k141_3265 | USW07202.1 putative RNA-dependent RNA polymerase [Plasmopara viticola lesion-associated ormycovirus 3] | 326 | 63.5 |  |
| SRR11547906 | k141_1919 | UUW20993.1 MAG: RNA-dependent RNA polymerase [Guiyang Paspalum thunbergii narna-like virus 1] | 3387 | 54 | Ophiocordyceps sinensis narnavirus 1 |
|  | k141_1529 | QNQ74063.1 RdRp [Plasmopara viticola lesion associated orfanplasmovirus 1] | 2892 | 38.8 | Ophiocordyceps sinensis narnavirus 2 |
|  | k141_870 | AZT88624.1 RNA-dependent RNA polymerase [Ophiocordyceps sinensis mitovirus 2] | 1256 | 97.6 | Ophiocordyceps sinensis mitovirus 2 |
|  | k141_1334 | USW07212.1 hypothetical protein [Erysiphe lesion-associated ormycovirus 2] | 949 | 32.8 | Ophiocordyceps sinensis ormycovirus 1 |
|  | k141_792 | AZT88624.1 RNA-dependent RNA polymerase [Ophiocordyceps sinensis mitovirus 2] | 917 | 96.4 |  |
|  | k141_177 | USW07207.1 putative RNA-dependent RNA polymerase [Erysiphe lesion-associated ormycovirus 2] | 708 | 46.4 |  |
|  | k141_1443 | PNF33053.1 Retrovirus-related Pol polyprotein from transposon TNT 1-94 [Cryptotermes secundus] | 660 | 61.2 |  |
|  | k141_1391 | AHF48631.1 RNA-dependent RNA polymerase [Sclerotinia sclerotiorum mitovirus 15] | 510 | 45.4 | Ophiocordyceps sinensis mitovirus 3 |
|  | k141_1782 | AHF48631.1 RNA-dependent RNA polymerase [Sclerotinia sclerotiorum mitovirus 15] | 396 | 67.2 |  |
| SRR11547905 | k141_126 | USW07203.1 hypothetical protein [Plasmopara viticola lesion-associated ormycovirus 3] | 438 | 44.8 | Ophiocordyceps sinensis ormycovirus 1 |
| SRR11547910 | k141_6005 | UUW20993.1 MAG: RNA-dependent RNA polymerase [Guiyang Paspalum thunbergii narna-like virus 1] | 3200 | 54 | Ophiocordyceps sinensis narnavirus 1 |
|  | k141_5342 | USW07204.1 putative RNA-dependent RNA polymerase [Erysiphe lesion-associated ormycovirus 3] | 468 | 49 | Ophiocordyceps sinensis ormycovirus 1 |
|  | k141_329 | USW07202.1 putative RNA-dependent RNA polymerase [Plasmopara viticola lesion-associated ormycovirus 3] | 432 | 51.7 |  |
|  | k141_3867 | GBP47781.1 Retrovirus-related Pol polyprotein from type-2 retrotransposable element R2DM; Endonuclease [Eumeta japonica] | 424 | 66.2 |  |
|  | k141_4550 | PNF33053.1 Retrovirus-related Pol polyprotein from transposon TNT 1-94 [Cryptotermes secundus] | 301 | 61.4 |  |
