# Supplementary material for: Unveiling mycoviral diversity in Ophiocordyceps sinensis through transcriptome analyses
Source: Front Microbiol. 2024 Nov 25;15:1493365. doi: 10.3389/fmicb.2024.1493365 (PMC11625762; doi:10.3389/fmicb.2024.1493365)
Supplement: Supplementary Table S9 — Detailed information on contigs obtained from 2 different samples by analyzing the PRJNA600609. [file Table_9.docx]

Table S9 Detailed information on contigs obtained from 2 different samples by analyzing the PRJNA600609.

| SRA ID | contig | protein description | Length (nt) | Ident (%) | Name of putative virus |
| --- | --- | --- | --- | --- | --- |
| SRR10878126 | k141_2511 | NP_044946.1 capsid polyprotein, partial [Drosophila C virus] | 3797 | 98.3 |  |
|  | k141_2696 | AAA46514.1 env protein [Murine leukemia virus] | 3212 | 89.7 |  |
|  | k141_297 | UYL95443.1 MAG: RNA-dependent RNA polymerase [Hulunbuir Botou tick virus 5] | 2888 | 52.1 | Ophiocordyceps ourmiavirus A |
|  | k141_1942 | USW07207.1 putative RNA-dependent RNA polymerase [Erysiphe lesion-associated ormycovirus 2] | 2470 | 46.4 | Ophiocordyceps sinensis ormycovirus 1 |
|  | k141_874 | USW07212.1 hypothetical protein [Erysiphe lesion-associated ormycovirus 2] | 2116 | 34.2 | Ophiocordyceps sinensis ormycovirus 1 |
|  | k141_222 | AMK06419.1 putative envelope polyprotein [Murine leukemia virus CasE#1] | 1815 | 92.2 |  |
|  | k141_136 | QNQ74063.1 RdRp [Plasmopara viticola lesion associated orfanplasmovirus 1] | 1103 | 39.7 | Ophiocordyceps sinensis narnavirus 2 |
|  | k141_1625 | BAD98609.1 env polyprotein [Murine leukemia virus] | 961 | 100 |  |
|  | k141_1224 | UYS78442.1 replicase polyprotein [Drosophila C virus] | 913 | 98.7 |  |
|  | k141_1120 | AXK59819.1 putative gag protein [Xenotropic murine leukemia virus] | 745 | 100 |  |
|  | k141_157 | AAA66622.1 reverse transcriptase, partial [Moloney murine leukemia virus] | 634 | 91 |  |
|  | k141_68 | AAA66622.1 reverse transcriptase, partial [Moloney murine leukemia virus] | 564 | 98.4 |  |
|  | k141_2232 | NP_044945.1 replicase polyprotein [Drosophila C virus] | 477 | 99.4 |  |
|  | k141_2364 | P0DOG8.1 RecName: Full=Glyco-Gag protein; AltName: Full=Gross cell surface antigen; AltName: Full=glycosylated Pr80 gag; Short=gPr80 Gag; Short=gag-gPr80 [AKR (endogenous) murine leukemia virus] | 469 | 99.1 |  |
|  | k141_142 | UYS78442.1 replicase polyprotein [Drosophila C virus] | 466 | 100 |  |
|  | k141_753 | P0DOG8.1 RecName: Full=Glyco-Gag protein; AltName: Full=Gross cell surface antigen; AltName: Full=glycosylated Pr80 gag; Short=gPr80 Gag; Short=gag-gPr80 [AKR (endogenous) murine leukemia virus] | 465 | 70.5 |  |
|  | k141_1874 | QNQ74064.1 RdRp [Plasmopara viticola lesion associated orfanplasmovirus 2] | 355 | 37.7 | Ophiocordyceps sinensis narnavirus 2 |
|  | k141_2133 | AAG10201.1 polyprotein, partial [Murine leukemia virus] | 341 | 99.1 |  |
|  | k141_2028 | P0DOG8.1 RecName: Full=Glyco-Gag protein; AltName: Full=Gross cell surface antigen; AltName: Full=glycosylated Pr80 gag; Short=gPr80 Gag; Short=gag-gPr80 [AKR (endogenous) murine leukemia virus] | 330 | 85.3 |  |
|  | k141_2445 | prf\|\|0711245A protein gag/pol/env [Moloney murine leukemia virus] | 310 | 100 |  |
|  | k141_255 | AAA46522.1 p30 protein, partial [Murine leukemia virus] | 306 | 97 |  |
| SRR10878121 | k141_1079 | NP_044946.1 capsid polyprotein, partial [Drosophila C virus] | 4597 | 98.3 |  |
|  | k141_1572 | AAA46531.1 envelope polyprotein [Xenotropic murine leukemia virus] | 2448 | 97.5 |  |
|  | k141_2230 | USW07207.1 putative RNA-dependent RNA polymerase [Erysiphe lesion-associated ormycovirus 2] | 2343 | 46.5 | Ophiocordyceps sinensis ormycovirus 1 |
|  | k141_575 | USW07212.1 hypothetical protein [Erysiphe lesion-associated ormycovirus 2] | 2118 | 34.1 | Ophiocordyceps sinensis ormycovirus 1 |
|  | k141_1420 | AAO61196.1 envelope polyprotein [Amphotropic murine leukemia virus] | 1448 | 100 |  |
|  | k141_2469 | BAD98609.1 env polyprotein [Murine leukemia virus] | 1210 | 99.5 |  |
|  | k141_1930 | QNQ74063.1 RdRp [Plasmopara viticola lesion associated orfanplasmovirus 1] | 1013 | 42.7 | Ophiocordyceps sinensis narnavirus 2 |
|  | k141_1542 | ADR80027.1 putative gag protein [Murine leukemia virus N417] | 778 | 91.3 |  |
|  | k141_820 | AFB82683.1 putative glyco-gag protein, partial [Xenotropic murine leukemia virus] | 588 | 94.8 |  |
|  | k141_1789 | AAC55820.1 reverse transcriptase, partial [Murine leukemia virus] | 586 | 99.5 |  |
|  | k141_331 | QNQ74066.1 RdRp [Plasmopara viticola lesion associated orfanplasmovirus 4] | 550 | 46.5 | Ophiocordyceps sinensis narnavirus 2 |
|  | k141_353 | ACY30461.1 putative gag polyprotein, partial [Xenotropic MuLV-related virus] | 506 | 95.8 |  |
|  | k141_32 | AAG10201.1 polyprotein, partial [Murine leukemia virus] | 474 | 100 |  |
|  | k141_321 | prf\|\|0711245A protein gag/pol/env [Moloney murine leukemia virus] | 423 | 97.3 |  |
|  | k141_875 | QEQ50987.1 replicase polyprotein [Drosophila C virus] | 365 | 100 |  |
|  | k141_1384 | AAA50476.1 putative, partial [Murine leukemia virus] | 334 | 100 |  |
|  | k141_1436 | QEQ50987.1 replicase polyprotein [Drosophila C virus] | 304 | 97.8 |  |
